# Genetic Profiling of Acute and Chronic Leukemia via Next-Generation Sequencing: Current Insights and Future Perspectives

**DOI:** 10.3390/hematolrep17020018

**Published:** 2025-03-28

**Authors:** Laras Pratiwi, Fawzia Hanum Mashudi, Mukti Citra Ningtyas, Henry Sutanto, Pradana Zaky Romadhon

**Affiliations:** 1 Internal Medicine Study Program, Department of Internal Medicine, Faculty of Medicine, Universitas Airlangga, Surabaya 60132, Indonesia; laras.pratiwi-2022@fk.unair.ac.id (L.P.); fawzia.hanum.mashudi-2022@fk.unair.ac.id (F.H.M.); mukti.citra.ningtyas-2022@fk.unair.ac.id (M.C.N.); 2 Department of Internal Medicine, Dr. Soetomo General Academic Hospital, Surabaya 60286, Indonesia; 3 Division of Hematology and Medical Oncology, Department of Internal Medicine, Faculty of Medicine, Universitas Airlangga, Surabaya 60132, Indonesia; 4 Department of Internal Medicine, Airlangga University Hospital, Surabaya 60115, Indonesia

**Keywords:** acute myeloid leukemia, acute lymphoblastic leukemia, chronic lymphocytic leukemia, chronic myeloid leukemia, genetic profiling, polymerase chain reaction, next-generation sequencing, clinical oncology

## Abstract

Leukemia is a heterogeneous group of hematologic malignancies characterized by distinct genetic and molecular abnormalities. Advancements in genomic technologies have significantly transformed the diagnosis, prognosis, and treatment strategies for leukemia. Among these, next-generation sequencing (NGS) has emerged as a powerful tool, enabling high-resolution genomic profiling that surpasses conventional diagnostic approaches. By providing comprehensive insights into genetic mutations, clonal evolution, and resistance mechanisms, NGS has revolutionized precision medicine in leukemia management. Despite its transformative potential, the clinical integration of NGS presents challenges, including data interpretation complexities, standardization issues, and cost considerations. However, continuous advancements in sequencing platforms and bioinformatics pipelines are enhancing the reliability and accessibility of NGS in routine clinical practice. The expanding role of NGS in leukemia is paving the way for improved risk stratification, targeted therapies, and real-time disease monitoring, ultimately leading to better patient outcomes. This review highlights the impact of NGS on leukemia research and clinical applications, discussing its advantages over traditional diagnostic techniques, key sequencing approaches, and emerging challenges. As precision oncology continues to evolve, NGS is expected to play an increasingly central role in the diagnosis and management of leukemia, driving innovations in personalized medicine and therapeutic interventions.

## 1. Introduction

Leukemia is a hematologic malignancy characterized by the uncontrolled proliferation of abnormal white blood cells originating from the bone marrow. It is broadly classified into four main types based on the cell lineage affected and the disease progression rate: acute lymphoblastic leukemia (ALL), acute myeloid leukemia (AML), chronic lymphocytic leukemia (CLL), and chronic myeloid leukemia (CML) (Table 1) [1]. Acute leukemias (ALL and AML) are characterized by rapid disease progression due to the accumulation of immature, dysfunctional blast cells, whereas chronic leukemias (CLL and CML) progress more slowly, often allowing for longer survival without immediate treatment [2,3,4,5]. ALL primarily affects lymphoid progenitor cells and is the most common leukemia in children, although it can occur in adults. It is further subclassified based on immunophenotyping into B-cell or T-cell ALL [4]. AML, on the other hand, arises from myeloid precursors and is the most prevalent acute leukemia in adults. It is classified into subtypes based on genetic and morphologic features, including recurrent mutations such as *FLT3*, *NPM1*, and *IDH1/2* [2]. CLL is a slow-growing leukemia affecting mature B lymphocytes and is predominantly found in older adults. It is often asymptomatic in early stages but can progress to a more aggressive form, requiring targeted therapy [5]. CML is defined by the presence of the *BCR::ABL1* fusion gene, resulting from the Philadelphia chromosome translocation, which drives the uncontrolled proliferation of myeloid cells [6]. The introduction of tyrosine kinase inhibitors (TKIs) has dramatically improved CML prognosis [7]. The classification of leukemia continues to evolve with advancements in molecular profiling, enabling better risk stratification and personalized treatment approaches [8,9].

Molecular profiling has become an essential tool in the diagnosis and treatment of leukemia, offering detailed insights into the genetic and epigenetic landscape of the disease [10,11]. Unlike conventional diagnostic methods, which primarily rely on cytogenetics, flow cytometry, and histopathological evaluation, molecular profiling enables the identification of specific genetic mutations, chromosomal rearrangements, and epigenetic modifications that define leukemia subtypes [11]. These insights have significantly improved risk stratification, guiding clinicians in selecting appropriate treatment strategies tailored to individual patients. For instance, genomic sequencing has revealed mutations in genes such as *FLT3*, *NPM1*, and *TP53*, which influence prognosis and therapeutic response in AML [12,13]. The integration of molecular profiling in leukemia treatment has also paved the way for targeted therapies, significantly improving patient outcomes [14]. The discovery of the *BCR::ABL1* fusion gene in CML, for example, led to the development of TKIs, such as imatinib, which have revolutionized CML management [15]. Similarly, the identification of *IDH1/2* and *FLT3* mutations in AML has resulted in the development of small-molecule inhibitors targeting these aberrations [16]. These advancements exemplify the shift toward precision medicine, where treatment regimens are customized based on the molecular characteristics of the leukemia subtype rather than a one-size-fits-all approach [17]. Moreover, molecular profiling facilitates real-time disease monitoring and early detection of relapse. Minimal residual disease (MRD) assessment using next-generation sequencing (NGS) and real-time polymerase chain reaction (PCR) techniques allows for the identification of residual leukemic cells that are undetectable by traditional methods [18,19]. This capability is particularly crucial in guiding post-remission therapy decisions and adjusting treatment intensity to prevent relapse [20,21]. The ability to track clonal evolution through serial genomic profiling further enhances personalized treatment approaches, as emerging resistance mutations can be identified and alternative therapies can be considered in a timely manner [22,23]. Despite these significant advancements, challenges remain in the widespread clinical implementation of molecular profiling. The high cost of sequencing technologies, the need for specialized bioinformatics expertise, and the complexity of interpreting vast genomic data pose barriers to routine clinical use. However, continuous advancements in sequencing efficiency, the integration of artificial intelligence in data interpretation, and efforts to standardize molecular testing protocols are expected to improve accessibility and reliability [23,24].

Here, we aim to explore the transformative role of NGS in the diagnosis, classification, and treatment of leukemia, with a particular focus on ALL, AML, CLL, and CML. By comparing NGS with conventional diagnostic methods, this review highlights its advantages in detecting genetic mutations, monitoring minimal residual disease, and guiding targeted therapies. Additionally, it examines the challenges associated with NGS implementation in clinical practice and discusses future directions for integrating this technology into routine leukemia management. Through this comprehensive analysis, the review aims to provide insights into how NGS is shaping the future of precision medicine in leukemia treatment.

## 2. Conventional Diagnostic Methods in Leukemia

### 2.1. Bone Marrow Aspiration and Biopsy

**Figure 1:**
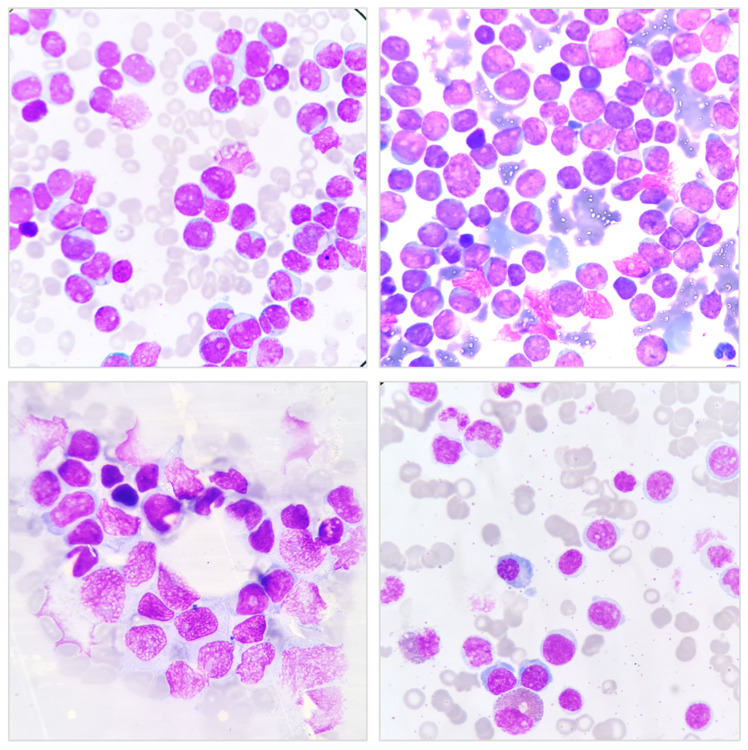
Bone Marrow Aspiration (BMA) Smear Showing Hematopoietic Cells. Representative microscopic images of a BMA smear stained with Wright–Giemsa stain. The images display various hematopoietic cells, including immature and mature forms, with characteristic nuclear and cytoplasmic features. The purple-stained nuclei contrast against the lighter cytoplasm, aiding in the differentiation of cell lineages. These images are essential for evaluating hematological disorders, including leukemia and other bone marrow pathologies.

Bone marrow aspiration and biopsy are essential diagnostic procedures in the evaluation of leukemia and other hematologic disorders [25]. Bone marrow aspiration involves extracting a liquid sample from the marrow, typically from the iliac crest, to assess cellular morphology, lineage involvement, and blast percentage (Figure 1) [26]. On the other hand, bone marrow biopsy provides a core tissue sample, allowing for the evaluation of marrow architecture, fibrosis, and cellularity. These procedures are frequently performed together to maximize diagnostic accuracy, particularly in cases where aspiration yields insufficient or inconclusive material [26]. Bone marrow aspiration is particularly useful in diagnosing acute leukemias, as it provides clear cytological details of blast morphology, essential for leukemia classification. In cases of chronic leukemias, such as CML or CLL, aspiration allows for the enumeration of abnormal cells and the detection of characteristic cytogenetic abnormalities [27]. However, one major limitation of aspiration is the potential for a “dry tap”, which occurs in conditions with increased marrow fibrosis, such as myelofibrosis or heavily infiltrated leukemic marrow [28,29]. In such cases, bone marrow biopsy becomes invaluable, as it provides a structural overview of the marrow and enables the detection of focal lesions or abnormal infiltration patterns [30]. Despite being the gold standard for leukemia diagnosis, bone marrow aspiration and biopsy are invasive procedures that require skilled personnel to ensure proper sample collection and minimize complications. Discomfort and minor bleeding are common, and in rare cases, infection or hemorrhage may occur [25]. Additionally, sample adequacy can impact diagnostic accuracy, as suboptimal samples may not fully reflect the disease burden. To improve diagnostic efficiency, these procedures are increasingly complemented by molecular and flow cytometric analyses, which provide deeper insights into leukemia pathophysiology and guide targeted treatment strategies [31,32].

### 2.2. Cytogenetics and Karyotyping

Cytogenetics and karyotyping play a crucial role in the diagnosis and prognostic evaluation of leukemia by identifying chromosomal abnormalities that contribute to disease pathogenesis [33,34]. Karyotyping, a technique that visualizes entire chromosome sets, helps detect numerical and structural chromosomal alterations, including translocations, deletions, duplications, and aneuploidies [35]. These chromosomal aberrations serve as essential biomarkers for risk stratification and treatment planning in various leukemia subtypes. For instance, in CML, the translocation *t(9;22)(q34;q11)*, known as the Philadelphia chromosome, results in the *BCR::ABL1* fusion gene, which drives leukemogenesis and is a hallmark of the disease [36]. The identification of this translocation has led to the development of TKIs, such as imatinib, which have transformed CML treatment and significantly improved patient survival. In AML, karyotyping is a key prognostic tool, as specific chromosomal translocations define subgroups with distinct treatment responses and survival outcomes [37]. For example, the *t(8;21)(q22;q22)* translocation is associated with a favorable prognosis, while complex karyotypes involving three or more chromosomal abnormalities correlate with poor outcomes [38]. Cytogenetic risk stratification is integral to AML treatment decisions, as patients with favorable cytogenetics may benefit from standard chemotherapy, whereas those with high-risk karyotypes may require allogeneic stem cell transplantation [39,40]. Similarly, in ALL, cytogenetic abnormalities such as *t(12;21)(p13;q22)*, which result in the *ETV6::RUNX1* fusion, indicate a favorable prognosis, whereas the presence of t(9;22) confers a poor prognosis and necessitates targeted therapy with TKIs [41,42]. Despite its diagnostic and prognostic value, conventional karyotyping has limitations, including the requirement for dividing cells, which may lead to unsuccessful metaphase analysis in cases with low mitotic index. Additionally, karyotyping has limited resolution and may miss submicroscopic genetic alterations [43]. As a result, fluorescence in situ hybridization (FISH) and molecular techniques, such as next-generation sequencing (NGS), are increasingly used alongside cytogenetics to improve diagnostic accuracy. Nevertheless, karyotyping remains a cornerstone in leukemia diagnostics, as it provides a comprehensive overview of chromosomal abnormalities, informs risk stratification, and guides therapeutic decisions [44].

### 2.3. Fluorescence In Situ Hybridization (FISH)

Fluorescence in situ hybridization (FISH) is a molecular cytogenetic technique widely used in leukemia diagnostics for detecting specific genetic abnormalities, such as gene fusions, deletions, and amplifications [45]. Unlike conventional karyotyping, which requires dividing cells for metaphase analysis, FISH can be performed on interphase nuclei, allowing for the detection of chromosomal abnormalities even in non-proliferating cells. This makes FISH particularly valuable for identifying recurrent cytogenetic alterations associated with different leukemia subtypes [46]. For instance, in CML, FISH is used to detect the *BCR::ABL1* fusion gene resulting from the Philadelphia chromosome translocation, a defining characteristic of the disease [47]. Similarly, in AML, FISH plays a crucial role in identifying translocations such as *t(8;21)*, *inv(16)*, and *t(15;17)*, which have significant prognostic and therapeutic implications [2,48]. In CLL, FISH is instrumental in detecting common genetic abnormalities, including deletions at *13q14*, *17p13* (*TP53*), and *11q22* (*ATM*), as well as trisomy 12. The detection of these abnormalities provides crucial prognostic information, as *TP53* and *ATM* deletions are associated with poor treatment response and aggressive disease progression. Studies have shown that FISH significantly increases the detection rate of cytogenetic abnormalities in CLL, particularly in cases with normal karyotypes, and is essential for refining risk stratification and guiding targeted therapy [49,50,51]. Moreover, FISH has been used to complement standard cytogenetic techniques in cases where conventional karyotyping fails to yield sufficient metaphases, thereby enhancing the overall diagnostic yield [52]. Beyond its diagnostic applications, FISH is also valuable for monitoring MRD and assessing treatment response in leukemia patients. In CML, for example, FISH is often used in combination with quantitative PCR to track the persistence or recurrence of *BCR::ABL1* transcripts following TKI therapy. Comparative studies have shown that while PCR is more sensitive for MRD detection, FISH remains a reliable method for assessing disease burden, particularly in resource-limited settings where PCR may not be readily available [53,54].

Despite its advantages, FISH has some limitations, including its reliance on pre-designed probes that target specific genetic abnormalities. This means that FISH cannot comprehensively assess all potential chromosomal aberrations in a single assay, necessitating the use of complementary techniques such as NGS for a more extensive genomic analysis. Furthermore, while FISH provides high sensitivity for known cytogenetic lesions, it does not detect point mutations or small insertions and deletions, which may also have prognostic significance in leukemia. Nonetheless, as an integral part of leukemia diagnostics, FISH continues to provide critical insights into genetic alterations, complementing other cytogenetic and molecular techniques to improve disease classification, prognostication, and treatment selection [55].

### 2.4. Polymerase Chain Reaction (PCR)

Polymerase chain reaction (PCR) is a powerful molecular diagnostic tool used in leukemia to identify specific genetic mutations associated with disease pathogenesis, prognosis, and treatment response [19,56]. By amplifying targeted DNA or RNA sequences, PCR enables the detection of mutations such as *BCR::ABL1* in CML, *NPM1* in AML, and *IDH1/2* mutations in various leukemia subtypes. These genetic alterations serve as biomarkers for risk stratification and personalized therapy, making PCR an essential component of leukemia diagnostics [57,58]. Several types of PCR techniques are used in leukemia research and clinical practice, each offering unique advantages [59]. Conventional PCR is commonly employed for the qualitative detection of fusion genes, such as *PML::RARA* in acute promyelocytic leukemia (APL), providing rapid confirmation of molecular abnormalities [60,61]. However, real-time quantitative PCR (RT-qPCR) has become the preferred method for monitoring MRD in leukemia patients. RT-qPCR allows for the quantification of leukemia-associated transcripts, such as *BCR::ABL1*, with high sensitivity, enabling the detection of disease recurrence at very low levels. This method has been particularly useful in assessing treatment response and guiding therapeutic decisions in CML patients undergoing tyrosine kinase inhibitor therapy [62,63]. Another variant, reverse transcription PCR (RT-PCR), is widely used for detecting gene fusions and aberrant transcript expression in leukemia [63]. RT-PCR is instrumental in diagnosing *BCR::ABL1* fusion variants and other translocations in ALL, such as *ETV6::RUNX1* and *MLL::AF4* [63,64]. Next, nested PCR (NPCR) enhances sensitivity by using two successive rounds of amplification, improving the detection of low-abundance mutations in leukemia patients [65,66]. Interestingly, a study compared the sensitivity of NPCR and RT-qPCR in detecting genetic alterations in acute leukemia patients. It showed that RT-qPCR was more sensitive than NPCR in detecting these alterations. Specifically, RT-qPCR identified 23 cases of *BCR::ABL1* and 9 cases of *TCF3::PBX1* in ALL patients, whereas detection rates for *FLT3-ITD*, *RUNX1::RUNX1T1*, *PML::RARA*, and *CBFB::MYH11* in AML patients were also more efficiently captured by RT-qPCR. The study concluded that RT-qPCR is the superior method for identifying genetic alterations in acute leukemia [67]. Meanwhile, droplet digital PCR (ddPCR) is an emerging technique that offers ultra-sensitive mutation detection by partitioning a sample into thousands of droplets and analyzing them individually. This technology is increasingly used in leukemia research for precise quantification of mutant allele fractions and MRD assessment. Compared to conventional PCR methods, ddPCR provides absolute quantification without the need for standard curves, making it a promising tool for refining leukemia diagnostics and treatment monitoring [68,69]. Despite its advantages, PCR has limitations, including the potential for contamination, false-positive results, and the inability to detect unknown mutations. Additionally, while PCR is highly sensitive for targeted genetic alterations, it does not provide a comprehensive genomic overview, necessitating the integration of NGS for a more complete leukemia mutational profile. Nonetheless, PCR remains a cornerstone of leukemia diagnostics, offering rapid, reliable, and cost-effective detection of clinically relevant mutations that guide treatment decisions [70,71,72].

### 2.5. Flow Cytometry

Flow cytometry is a widely used immunophenotyping technique that plays a crucial role in leukemia diagnosis, classification, and prognosis. This technology enables the rapid and precise characterization of hematologic malignancies by identifying specific surface and intracellular markers expressed by leukemic cells. By using fluorescently labeled antibodies directed against lineage-specific antigens, flow cytometry allows for the differentiation of leukemia subtypes based on their immunophenotypic profiles. Compared to traditional microscopic examination and cytochemistry, flow cytometry offers higher sensitivity and specificity, making it a cornerstone in modern leukemia diagnostics [73,74]. One of the primary advantages of flow cytometry in leukemia classification is its ability to distinguish between AML and ALL [75]. AML cells typically express markers such as CD13, CD33, and CD117, whereas ALL is characterized by the presence of B-cell markers (CD19, CD22, CD79a) or T-cell markers (CD3, CD7, CD5) [73]. Moreover, flow cytometry can identify mixed phenotype acute leukemia (MPAL), a rare subtype expressing both myeloid and lymphoid markers, which has distinct therapeutic implications [76]. Beyond initial diagnosis, flow cytometry is essential for assessing MRD after chemotherapy. MRD refers to the presence of residual leukemic cells that are undetectable by conventional microscopy but can be quantified using highly sensitive multiparametric flow cytometry. MRD levels have strong prognostic value, guiding treatment decisions and risk stratification. For example, in ALL, patients with undetectable MRD after induction therapy have a significantly lower risk of relapse compared to those with persistent MRD [18,77].

Despite its advantages, flow cytometry has some limitations, including the need for fresh samples and the complexity of data interpretation. Variability in antigen expression due to treatment effects or disease evolution may lead to challenges in MRD detection. Additionally, while flow cytometry provides valuable immunophenotypic data, it does not detect genetic mutations, necessitating complementary molecular techniques such as PCR and NGS for a more comprehensive leukemia assessment [78,79,80]. The comparison of several diagnostic methods in acute and chronic leukemia is shown in Table 2.

### 2.6. Other Limitations of Conventional Methods

Despite significant advancements in leukemia diagnostics, conventional methods such as karyotyping, FISH, PCR, and flow cytometry have notable limitations that have driven the adoption of NGS. One of the primary shortcomings of traditional cytogenetics and molecular techniques is incomplete genomic coverage. Karyotyping, while useful for detecting large-scale chromosomal abnormalities, fails to identify submicroscopic mutations and cryptic translocations that can significantly impact leukemia prognosis and treatment decisions [81]. Similarly, FISH is limited to targeted analysis of predefined genetic rearrangements, making it ineffective for comprehensive genomic profiling [82]. PCR-based assays are highly sensitive but can only detect known mutations, necessitating multiple assays for comprehensive leukemia classification [83]. Another major limitation of conventional diagnostic approaches is their lower sensitivity compared to targeted NGS, particularly in the detection of MRD and subclonal mutations. Traditional Sanger sequencing, for example, has a detection threshold of around 15–20% variant allele frequency, meaning low-level mutations may go undetected. This limitation is particularly concerning in leukemia cases where early detection of emerging resistant clones is critical for treatment modifications [56]. Studies have shown that NGS-based approaches offer superior sensitivity, enabling the identification of low-abundance mutations that may contribute to disease relapse [84]. Additionally, targeted NGS panels have demonstrated higher concordance rates with traditional cytogenetics while uncovering additional clinically relevant mutations missed by standard methods [85]. Conventional diagnostic methods also lack the ability to provide insights into clonal evolution, a critical factor in leukemia progression and therapeutic resistance. Leukemia is a highly heterogeneous disease that evolves over time, often acquiring new mutations that contribute to treatment resistance and disease relapse [2,86]. Standard techniques such as FISH and PCR offer only a static snapshot of the disease at a given time, failing to capture the dynamic genetic changes that occur throughout treatment. In contrast, NGS enables longitudinal monitoring of clonal evolution by identifying emerging subclones and tracking mutational shifts during therapy. This ability to detect and characterize clonal diversity is essential for adapting treatment strategies and improving patient outcomes [87,88]. The transition from conventional diagnostics to NGS has been driven by these limitations, as NGS provides a more comprehensive, sensitive, and dynamic approach to leukemia characterization [89].

## 3. Basics, Techniques, and Procedures of NGS

### 3.1. Basics of NGS

NGS has revolutionized the field of molecular diagnostics, offering high-throughput, comprehensive genomic analysis that surpasses traditional sequencing techniques. Unlike Sanger sequencing, which can only analyze one gene at a time, NGS allows for the simultaneous sequencing of millions of DNA fragments, providing a more efficient and cost-effective method for detecting genetic variations [90]. This advancement has been particularly transformative in leukemia research and clinical management, enabling the identification of novel driver mutations, understanding clonal evolution, and refining risk stratification [87,91,92]. One of the primary advantages of NGS is its ability to detect a wide spectrum of genetic alterations, including single-nucleotide variants (SNVs), insertions and deletions (indels), copy number variations (CNVs), and gene fusions, all within a single assay. This capability is particularly important for leukemia subtypes such as AML and ALL, where genetic heterogeneity influences treatment outcomes [93,94]. By providing detailed molecular profiles, NGS facilitates personalized medicine approaches, allowing clinicians to tailor treatments based on the genetic makeup of an individual’s leukemia [95,96]. Sensitivity is a crucial aspect when comparing NGS to Sanger sequencing. Studies have shown that Sanger sequencing has a variant allele frequency (VAF) detection threshold of approximately 15–20%, meaning it can only reliably detect mutations when they are present in at least 15% of the DNA sample [56]. In contrast, NGS can detect mutations with a VAF as low as 1–5%, significantly enhancing its ability to identify rare mutations and clonal heterogeneity [97]. This is particularly relevant in leukemia, where early detection of emerging resistant clones is critical for adjusting treatment before full-blown relapse occurs. Another advantage of NGS is its ability to provide deeper coverage, meaning it sequences the same genomic region multiple times to improve accuracy [98].

Beyond diagnostics, NGS plays a crucial role in monitoring MRD and tracking clonal evolution over time. Traditional MRD assessment methods, such as flow cytometry and PCR, have limitations in sensitivity and specificity, whereas NGS enables the detection of residual leukemic clones at much lower thresholds. This is particularly relevant in guiding treatment decisions, as early detection of relapse-associated mutations can inform adjustments in therapy, potentially improving patient outcomes [94,99]. Another key advantage of NGS is its ability to uncover previously unrecognized mutations that contribute to treatment resistance. Deep sequencing ensures that even minor subclonal mutations are detected, which is essential for understanding the genetic complexity of leukemia. For example, in CML, NGS can detect compound mutations in the *BCR::ABL1* kinase domain that contribute to TKI resistance, whereas Sanger sequencing often fails to distinguish between polyclonal and compound mutations [100,101]. In CLL, NGS has identified mutations in genes such as *TP53*, *NOTCH1*, and *SF3B1*, which are associated with poor prognosis and resistance to conventional chemotherapy. This information allows for more precise risk stratification and the selection of alternative therapeutic strategies, such as targeted inhibitors [87,96].

### 3.2. Techniques of NGS

NGS encompasses several distinct approaches, each offering unique advantages for leukemia research and clinical diagnostics (Table 3). Whole-genome sequencing (WGS) is the most comprehensive method, as it analyzes the entire genome, including both coding and non-coding regions [102]. This approach is particularly useful for discovering novel mutations, detecting structural variations, and identifying non-coding regulatory elements that may contribute to leukemia pathogenesis [103]. However, the extensive data generated by WGS, along with its high cost and computational demands, have limited its routine clinical use. Despite these challenges, WGS remains a valuable tool for research and precision medicine initiatives, as it provides an unbiased view of the genetic landscape of leukemia [104]. Whole-exome sequencing (WES) offers a more targeted approach by focusing only on the protein-coding regions of the genome, which comprise approximately 1–2% of the entire genome but contain the majority of known disease-causing mutations [102]. WES is a cost-effective alternative to WGS while still capturing clinically relevant mutations in leukemia-related genes [105]. It has been instrumental in identifying key mutations such as *NPM1*, *FLT3*, and *TP53*, which play crucial roles in leukemia prognosis and treatment response. Although WES does not provide insights into non-coding regions or large structural variations, it remains a widely used approach in both research and clinical settings due to its efficiency and high diagnostic yield [106].

Targeted gene panels are designed to sequence a predefined set of genes known to be associated with leukemia, providing a cost-effective and clinically applicable solution for mutation detection. These panels offer high sequencing depth, allowing for the identification of low-frequency mutations that may be missed by WES or WGS [102]. Targeted NGS panels are commonly used in clinical diagnostics to detect actionable mutations that can inform treatment decisions, such as *IDH1/2* and *DNMT3A* mutations in AML [107]. Moreover, they reduce data complexity and interpretation challenges compared to broader sequencing approaches, making them highly suitable for routine clinical applications [108]. RNA sequencing (RNA-Seq) is a specialized NGS approach that focuses on the transcriptome, allowing for the detection of fusion genes, alternative splicing events, and gene expression patterns in leukemia [109]. RNA-Seq is particularly useful for identifying oncogenic fusion transcripts, such as *BCR::ABL1* in CML or *ETV6::RUNX1* in ALL, which may not be detected by DNA-based sequencing methods. Additionally, RNA-Seq provides insights into gene expression profiles that can help classify leukemia subtypes and predict treatment responses. By capturing both known and novel gene fusions, RNA-Seq has become an indispensable tool for leukemia research and diagnostics [110,111,112]. The selection of an NGS approach depends on the specific clinical or research question, balancing cost, sequencing depth, and data complexity. While WGS provides the most comprehensive view, WES and targeted panels offer practical and cost-effective alternatives for clinical applications, and RNA-Seq complements these methods by providing transcriptomic insights [113].

### 3.3. Step-by-Step Procedures of NGS

**Figure 2:**
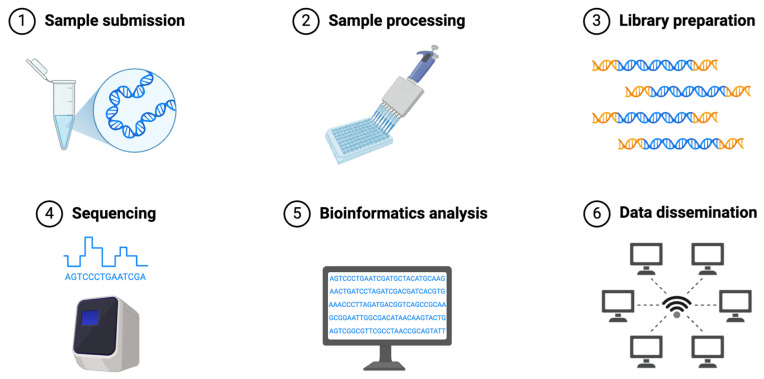
Common Workflow of Next-Generation Sequencing.

The NGS workflow consists of several key steps that ensure accurate and comprehensive genomic analysis in leukemia diagnostics (Figure 2) [114]. The process begins with sample preparation, during which DNA or RNA is extracted from sources such as bone marrow aspirates, peripheral blood, or biopsy samples. The quality and purity of nucleic acids are critical for the success of downstream applications, as degraded or contaminated samples can lead to sequencing artifacts or incomplete coverage [114]. In leukemia, high-quality sample extraction is essential for detecting mutations associated with disease progression and treatment resistance [115]. Following extraction, library preparation involves fragmenting DNA or RNA into small, uniform segments, which are then ligated to sequencing adapters and amplified. This step ensures that sequencing platforms can process the samples efficiently, maximizing coverage and minimizing sequencing bias [114]. Different approaches, such as amplicon-based enrichment or hybrid capture, are used depending on the NGS application. For targeted gene panels in leukemia, amplicon-based enrichment is commonly employed due to its high sensitivity in detecting known mutations with low variant allele frequencies [116].

The sequencing step utilizes high-throughput platforms such as Illumina, Oxford Nanopore, and PacBio, each offering distinct advantages (Table 4) [117]. Illumina sequencing, which is widely used in leukemia diagnostics, provides high accuracy and deep coverage, making it ideal for identifying single-nucleotide variants and small insertions or deletions [99,118]. Oxford Nanopore and PacBio long-read sequencing technologies offer additional capabilities, such as resolving complex structural variants and detecting full-length transcript isoforms in RNA-Seq [117]. These advancements enhance leukemia classification and aid in identifying novel fusion genes [118]. Once sequencing is complete, bioinformatics pipelines process the raw data, performing quality control, alignment to a reference genome, variant calling, and annotation. These pipelines use specialized software tools to filter out sequencing errors and distinguish true variants from background noise. Advanced computational approaches are employed to interpret structural variations, gene fusions, and alternative splicing events, which are critical for leukemia diagnosis [114]. For example, WES combined with RNA-Seq enables a comprehensive view of both genomic mutations and transcriptomic changes, improving diagnostic accuracy [114]. The final step in the NGS workflow is clinical interpretation, where identified variants are analyzed for their relevance to leukemia pathogenesis, prognosis, and therapeutic decision-making. This involves cross-referencing detected mutations with established databases such as the Catalogue of Somatic Mutations in Cancer (COSMIC) and ClinVar, which provide insights into the clinical significance of genetic alterations [119,120]. In leukemia, integrating NGS results with clinical and cytogenetic data allows for personalized treatment strategies, including the selection of targeted therapies based on specific driver mutations [95].

## 4. NGS in Acute Lymphoblastic Leukemia (ALL)

NGS plays a crucial role in the diagnosis, risk stratification, and treatment planning of ALL. NGS enables comprehensive genomic profiling, allowing for the identification of novel molecular subtypes, driver mutations, and chromosomal alterations that impact disease progression and therapeutic response [94]. NGS is particularly valuable in identifying cryptic genetic lesions that may not be detected by conventional cytogenetics, such as rare gene fusions or mutations in genes like *IKZF1*, *CRLF2*, and *JAK2*, which influence prognosis and treatment decisions [121]. Additionally, NGS helps in subclassifying ALL into molecularly distinct subtypes, such as Philadelphia chromosome-like (Ph-like) ALL, which requires targeted therapy [122].

Among the most critical genetic abnormalities detected by NGS in ALL are alterations in *IKZF1*, *ETV6::RUNX1*, and *CRLF2* (Table 5) [123,124]. *IKZF1* deletions are a significant genetic aberration in ALL, particularly in B-cell precursor ALL (BCP-ALL), where they are associated with poor prognosis and therapy resistance [124]. These deletions occur in approximately 10–15% of pediatric and adult BCP-ALL cases, with a higher prevalence in *BCR::ABL1*-positive and Ph-like ALL subtypes [125,126]. Mechanistically, *IKZF1* deletions contribute to leukemogenesis by disrupting normal Ikaros function, leading to impaired B-cell differentiation and enhanced leukemic cell proliferation [124]. Studies have shown that patients with *IKZF1* deletions exhibit lower event-free survival (EFS) and higher cumulative relapse rates compared to those without the deletion [127,128]. In pediatric BCP-ALL, a higher burden of *IKZF1* deletions (>1%) correlates with significantly reduced survival rates [129,130,131]. Furthermore, *IKZF1* deletions have been implicated in treatment resistance, particularly to cytarabine, due to reduced drug uptake mechanisms [132]. The *ETV6::RUNX1* fusion, resulting from the *t(12;21)(p13;q22)* translocation, is the most common genetic alteration in childhood BCP-ALL, accounting for approximately 20–25% of cases [133]. This fusion event occurs in utero and is considered an initiating mutation; however, it is insufficient to drive leukemia independently, necessitating additional genetic alterations for full leukemogenesis [134]. Studies in twins and mouse models have demonstrated that *ETV6::RUNX1* establishes a preleukemic clone that can persist for years before acquiring secondary mutations that lead to overt leukemia [135,136]. The fusion protein functions as an aberrant transcription factor that disrupts normal hematopoietic differentiation by interfering with RUNX1-mediated gene regulation, leading to transcriptional repression of key developmental genes [137,138]. Despite its role in leukemogenesis, *ETV6::RUNX1* is generally associated with a favorable prognosis, with affected patients responding well to standard chemotherapy protocols and exhibiting high survival rates [139]. However, relapse in *ETV6::RUNX1*-positive ALL remains a concern, often driven by additional genetic events such as deletions in *ETV6* or mutations in cell cycle regulators [41]. Meanwhile, *CRLF2* rearrangements are a significant genetic alteration in ALL, particularly within the Ph-like B-ALL subtype, a high-risk subgroup of B-ALL that may benefit from TKIs, where they occur in approximately 50% of cases [94,140]. These rearrangements most commonly result from either an *IGH-CRLF2* translocation or a *P2RY8-CRLF2* fusion, both of which lead to overexpression of the cytokine receptor-like factor 2 (*CRLF2*) and subsequent activation of the JAK-STAT signaling pathway [141]. Studies have shown that *CRLF2* rearrangements frequently co-occur with activating mutations in *JAK2*, as well as deletions or mutations in *IKZF1*, which contribute to leukemogenesis and poor treatment response [142]. Patients with *CRLF2* rearrangements have been observed to have a significantly increased risk of relapse and inferior survival outcomes compared to those without the alteration, with studies reporting a relapse-free survival rate of only 35.3% at four years [141,142]. Additionally, aberrant activation of downstream pathways such as PI3K/mTOR has been documented in *CRLF2*-rearranged ALL, highlighting potential therapeutic targets for treatment-resistant cases [143]. Recent studies suggest that patients with *CRLF2* rearrangements exhibit resistance to glucocorticoids, a key component of ALL therapy, but this resistance can be overcome with MEK or Akt inhibition, presenting new avenues for targeted treatment [140].

NGS has become an essential tool in risk stratification and treatment decision-making in ALL. The presence of high-risk mutations, such as those in *IKZF1*, *TP53*, or the RAS signaling pathway, influences treatment intensity and therapeutic choices [94]. For example, RAS pathway mutations, including *NRAS*, *KRAS*, and *PTPN11*, have been identified as potential drivers of ALL relapse, indicating the need for closer monitoring and possible early intervention with targeted agents [144]. The integration of NGS-based classifiers into clinical risk models has refined traditional prognostic scoring systems by incorporating genetic insights that were previously undetectable by conventional methods. This approach has led to the identification of very high-risk subgroups within T-ALL and B-ALL that require intensified therapy or novel treatment strategies [145]. By enabling early detection of mutations associated with treatment resistance, NGS has facilitated the development of precision medicine approaches in ALL, including the use of targeted inhibitors for kinase-activating lesions in Ph-like ALL [146]. Another impactful application of NGS in ALL is its role in MRD monitoring. MRD, defined as the presence of leukemic cells below the detection threshold of conventional microscopy, is a powerful prognostic indicator used for treatment stratification. NGS-based MRD assays provide greater sensitivity compared to flow cytometry and qPCR, allowing for the detection of one leukemic cell in a million normal cells [147]. This heightened sensitivity has enabled the identification of patients at risk of relapse despite achieving complete remission by conventional methods. Clinical trials have demonstrated that NGS-MRD outperforms multiparametric flow cytometry in detecting residual disease and predicting long-term outcomes, particularly in patients with B-ALL and T-ALL [148]. However, the prognostic value of NGS-based MRD remains an area of active research, with some studies suggesting that MRD positivity detected at ultra-low levels may not always correlate with an increased risk of relapse [149].

## 5. NGS in Acute Myeloid Leukemia (AML)

NGS has significantly enhanced the understanding of the genomic landscape of AML, revealing key driver mutations such as *FLT3*, *NPM1*, and *IDH1/2* (Table 6) [150]. The *FLT3* mutation, particularly the internal tandem duplication (FLT3-ITD), is one of the most prevalent alterations in AML, occurring in approximately 25–30% of cases [151]. It is associated with aggressive disease progression and poor prognosis [152]. Mutations in *NPM1*, a nucleophosmin gene, are found in around 30% of adult AML patients and are considered favorable prognostic markers in the absence of *FLT3-ITD* [153]. Patients with *NPM1* mutations but without *FLT3-ITD* have improved clinical outcomes, likely due to a more favorable response to chemotherapy and higher rates of complete remission [154,155]. In contrast, *NPM1* mutations co-occurring with *FLT3-ITD* neutralize the positive prognostic effect of *NPM1*, leading to a worse prognosis comparable to *FLT3-ITD*-only cases [156]. The adverse impact of *FLT3-ITD* is largely attributed to increased leukemic cell proliferation, resistance to apoptosis, and enhanced aggressiveness of the disease [157]. Studies have shown that in patients with *NPM1*-mutated AML, those lacking *FLT3-ITD* exhibit significantly longer disease-free and overall survival compared to those with both mutations [158]. The absence of *FLT3-ITD* allows *NPM1*-mutated AML to remain more responsive to standard induction chemotherapy, leading to better remission rates and prolonged survival. Consequently, *NPM1*-mutated, *FLT3-ITD*-negative AML is classified as a favorable-risk group in the European LeukemiaNet (ELN) guidelines, whereas *FLT3-ITD* positivity places patients in an intermediate- or adverse-risk category depending on the allelic ratio and co-occurring mutations [159].

Similarly, *IDH1* and *IDH2* mutations (Table 6) occur in 5–20% of AML cases and play a crucial role in leukemogenesis by altering DNA methylation and cellular metabolism [160]. These mutations affect key metabolic enzymes, leading to the production of the oncometabolite 2-hydroxyglutarate, which disrupts normal DNA methylation and inhibits differentiation of hematopoietic cells, thereby promoting leukemogenesis [161]. *IDH1* mutations, primarily affecting codon R132, are more frequently found in cytogenetically normal AML and often co-occur with *NPM1* mutations. *IDH2* mutations, particularly R140Q and R172K, are also associated with normal karyotype AML but tend to be mutually exclusive with *IDH1* mutations [161,162]. The prognostic impact of these mutations varies depending on the specific mutation and co-occurring genetic alterations. *IDH1* R132 mutations have been associated with poor prognosis in some studies, particularly when they occur in the absence of *FLT3-ITD* or *NPM1* mutations [163,164]. *IDH2* R172 mutations have also been linked to adverse outcomes, while *IDH2* R140Q mutations appear to have a more neutral or slightly favorable prognostic effect. Therapeutically, *IDH1* and *IDH2* inhibitors, such as ivosidenib and enasidenib, respectively, have been developed to target these mutations. These inhibitors work by restoring normal differentiation in leukemic cells and have shown clinical efficacy, particularly in relapsed or refractory AML [165]. Ongoing research is focused on understanding resistance mechanisms to IDH inhibitors, as leukemia stemness and co-occurring mutations, such as *RUNX1* and *RAS-RTK* pathway alterations, can drive resistance to these targeted therapies [166]. Overall, the identification of these mutations through NGS has refined AML classification, prognosis, and personalized treatment approaches. The impact of NGS on personalized therapy in AML is profound, particularly in the selection of targeted inhibitors. FLT3 inhibitors, such as midostaurin and gilteritinib, have shown clinical efficacy in FLT3-mutant AML by inhibiting aberrant tyrosine kinase signaling. Patients with *IDH1/2* mutations benefit from specific inhibitors like ivosidenib and enasidenib, which restore normal hematopoietic differentiation [167]. These targeted therapies, guided by NGS-based mutation profiling, have led to improved treatment outcomes and prolonged survival for AML patients. The incorporation of NGS into clinical workflows allows for the rapid detection of actionable mutations, ensuring the timely initiation of personalized treatment regimens [168]. Furthermore, NGS-based stratification has been instrumental in optimizing chemotherapy regimens, as patients harboring concurrent *NPM1* and *FLT3-ITD* mutations may require intensified therapeutic strategies [169].

Beyond diagnosis and treatment selection, NGS plays a critical role in monitoring clonal evolution and treatment resistance in AML. Leukemia is characterized by significant genetic heterogeneity, with subclonal populations evolving in response to therapy. NGS enables the longitudinal tracking of mutational changes, revealing the emergence of resistant clones that drive relapse [86]. For example, patients treated with FLT3 inhibitors may develop secondary mutations in the kinase domain, necessitating alternative therapeutic strategies [170]. Similarly, the persistence or acquisition of *IDH1/2* mutations following chemotherapy has been linked to disease relapse, underscoring the importance of dynamic molecular monitoring [171]. NGS-based MRD assessment allows for the detection of residual leukemic clones at ultra-low levels, providing a more sensitive approach to relapse prediction compared to traditional methods [172,173].

## 6. NGS in Chronic Lymphocytic Leukemia (CLL)

NGS has revolutionized the molecular characterization of CLL by identifying key mutations with significant prognostic and therapeutic implications [87]. Among these, *TP53*, *NOTCH1*, and *SF3B1* mutations play critical roles in disease progression and treatment resistance (Table 7) [174]. *TP53* mutations in CLL are among the most critical genetic alterations, as they are strongly associated with poor prognosis, rapid disease progression, and resistance to standard chemoimmunotherapy. These mutations occur in approximately 5–10% of newly diagnosed CLL cases but are found in over 40% of relapsed or refractory patients, highlighting their role in disease evolution [175]. The majority of *TP53* mutations are located within the DNA-binding domain and lead to loss of function, impairing the tumor suppressor’s ability to regulate apoptosis and cell cycle arrest. Notably, *TP53* mutations frequently co-occur with 17p deletions, where one allele is lost, and the remaining allele is mutated, further exacerbating disease aggressiveness [176]. However, *TP53* mutations can also occur independently of 17p deletion, albeit with similar negative prognostic implications. Studies have demonstrated that CLL patients harboring *TP53* mutations exhibit significantly shorter progression-free and overall survival compared to those without these mutations, even when treated with fludarabine-based regimens [177,178]. The presence of *TP53* mutations also predicts poor response to standard chemoimmunotherapy, necessitating the use of novel targeted agents such as Bruton’s tyrosine kinase (BTK) inhibitors (ibrutinib, acalabrutinib) and B-cell lymphoma 2 (BCL-2) inhibitors (venetoclax), which have demonstrated superior efficacy in *TP53*-mutated CLL [179,180]. Due to its strong prognostic and predictive value, *TP53* mutation testing is now recommended before initiating treatment in CLL patients, and repeated testing is advised before each line of therapy, as clonal evolution may lead to the emergence of new *TP53* mutations over time [181,182,183].

Meanwhile, *NOTCH1* mutations (Table 7) occur in approximately 10–15% of newly diagnosed cases and are increasing in prevalence in relapsed or refractory disease [184]. These mutations typically involve frameshift or nonsense alterations in exon 34, leading to the truncation of the PEST domain, which results in prolonged *NOTCH1* activation due to impaired degradation of the *NOTCH1* intracellular domain (NICD) [185]. The constitutive activation of *NOTCH1* signaling promotes leukemic cell survival, proliferation, and resistance to apoptosis, contributing to disease progression. Clinically, *NOTCH1* mutations are associated with an aggressive disease course, shorter overall survival, and reduced time to first treatment, making them a strong negative prognostic marker [186]. These mutations frequently co-occur with trisomy 12 and unmutated *IGHV*, both of which are indicative of a more aggressive CLL phenotype. Importantly, *NOTCH1* mutations have been linked to resistance to anti-CD20 monoclonal antibody therapies, such as rituximab and obinutuzumab, potentially explaining inferior responses to chemoimmunotherapy [187]. Moreover, studies suggest a strong association between *NOTCH1* mutations and an increased risk of Richter’s transformation, the progression of CLL into aggressive diffuse large B-cell lymphoma (DLBCL), which significantly worsens patient prognosis [188]. Given their clinical relevance, *NOTCH1* mutations are now considered an independent prognostic marker in CLL, and testing for these alterations is recommended for risk stratification and treatment decision-making. The development of *NOTCH1* inhibitors and γ-secretase inhibitors as potential therapeutic strategies is an active area of research, aiming to counteract the oncogenic effects of *NOTCH1* activation in CLL [189]. Next, *SF3B1* mutations occur in approximately 10–15% of cases and are increasing in frequency in advanced and treatment-refractory disease [190]. *SF3B1* encodes a core component of the spliceosome, which is essential for pre-mRNA splicing, and mutations in this gene lead to widespread alterations in RNA processing. These splicing defects contribute to CLL pathogenesis by disrupting normal gene expression and promoting leukemic cell survival [191,192]. Transcriptomic analyses have shown that *SF3B1* mutations affect multiple key pathways, including DNA damage response, telomere maintenance, and NOTCH signaling, further driving disease progression [193]. Clinically, *SF3B1* mutations are associated with an aggressive CLL phenotype, characterized by shorter treatment-free survival, poor response to fludarabine-based chemoimmunotherapy, and increased risk of disease progression [194]. Additionally, *SF3B1* mutations frequently co-occur with *del(11q)*, which is another high-risk genetic feature in CLL, further reinforcing their association with adverse clinical outcomes [195]. Given their prognostic significance, *SF3B1* mutations are now included in molecular risk stratification models for CLL [196]. While standard chemoimmunotherapy is less effective in patients with these mutations, targeted agents such as BTK inhibitors (e.g., ibrutinib) and BCL-2 inhibitors (e.g., venetoclax) have shown better clinical outcomes in *SF3B1*-mutated CLL [197,198].

Another crucial application of NGS in CLL is the detection of subclonal evolution and treatment resistance [199]. CLL is characterized by significant genetic heterogeneity, with multiple subclonal populations evolving under therapeutic pressure. NGS allows for the tracking of these subclones, revealing dynamic changes in the mutational landscape that contribute to disease relapse [199]. NGS has also significantly influenced therapy selection in CLL, particularly guiding the use of BTK and BCL-2 inhibitors. BTK inhibitors, such as ibrutinib and acalabrutinib, target the B-cell receptor signaling pathway and have shown efficacy in high-risk patients, including those with *TP53* aberrations. However, resistance often develops through acquired mutations in BTK (e.g., *C481S*) and *PLCG2*, which render the inhibitors less effective [200]. NGS enables early detection of these resistance-associated mutations, allowing for timely treatment adjustments. Similarly, BCL2 inhibitors like venetoclax are highly effective in *TP53*-mutated CLL, but resistance can emerge due to mutations in *BCL2* itself, leading to therapeutic failure [201]. NGS-based monitoring of these mutations has facilitated sequential therapy strategies, optimizing treatment outcomes [202]. Additionally, *TP53* and *NOTCH1* mutations have been implicated in disease progression despite targeted therapy, highlighting the need for continuous molecular monitoring [203]. The ability of NGS to detect emerging resistant clones before clinical relapse allows for preemptive treatment modifications, such as switching therapy or combining targeted agents to delay disease progression [204].

## 7. NGS in Chronic Myeloid Leukemia (CML)

**Figure 3:**
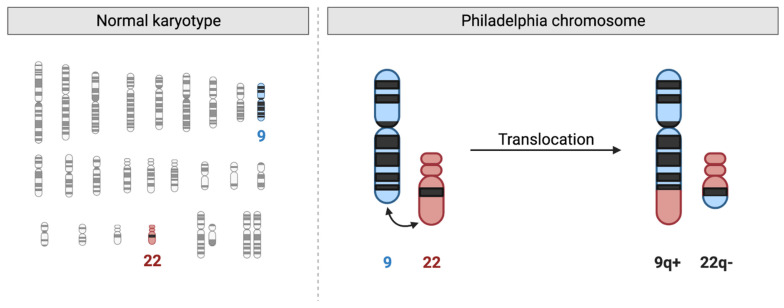
The Philadelphia Chromosome in Chronic Myeloid Leukemia (CML). On the left, a normal human karyotype is shown, highlighting chromosomes 9 (blue) and 22 (red). On the right, the reciprocal translocation is depicted, where part of chromosome 9q (*ABL1* gene) translocates to chromosome 22q, creating the shortened chromosome 22 (22q−, Philadelphia chromosome) and a reciprocal fusion on chromosome 9q (9q+). This translocation results in the *BCR::ABL1* fusion gene, which produces an oncogenic tyrosine kinase responsible for the pathogenesis of CML.

NGS has transformed the molecular characterization of CML by improving the detection of *BCR::ABL1* fusion variants and resistance mutations [205,206]. The *BCR::ABL1* fusion gene, resulting from the t(9;22) chromosomal translocation (Figure 3), is the hallmark of CML and drives disease pathogenesis through constitutive tyrosine kinase activity. While traditional molecular techniques such as qPCR are routinely used for *BCR::ABL1* transcript monitoring, they may fail to detect atypical or complex fusion variants [207]. NGS has enabled the identification of previously unrecognized *BCR::ABL1* isoforms, facilitating more precise molecular classification and guiding targeted therapy decisions [208]. Atypical *BCR::ABL1* mutations in CML represent rare but clinically significant genetic alterations that can impact disease progression and treatment response. While the classic *BCR::ABL1* fusion results from the *t(9;22)* translocation, atypical variants involve uncommon breakpoints, alternative splicing, or additional genetic rearrangements that can lead to unusual fusion transcripts. These atypical mutations often result in differential expression levels or altered tyrosine kinase activity, which may influence sensitivity to TKIs [209]. One example is the e6a2 fusion transcript, which is associated with an aggressive disease course and increased risk of blast crisis, potentially due to the fusion protein’s altered structural and functional properties [210]. Another rare transcript, e18a2, has been reported to cause diagnostic ambiguity due to its atypical splicing pattern, which complicates molecular detection and monitoring [211,212]. Fusion variants arise due to variations in the *BCR::ABL1* translocation, resulting in different fusion transcripts that can influence disease progression and response to TKIs. The most common fusion variants include e13a2 (b2a2) and e14a2 (b3a2), both encoding the p210 *BCR::ABL1* protein, which is characteristic of classical CML [213]. However, rarer fusion variants such as e13a3 (b2a3) and e14a3 (b3a3) have been identified, which may lead to difficulties in molecular detection using standard PCR assays and require alternative diagnostic techniques like FISH or sequencing [214,215]. Some fusion variants, such as *e19a2*, are associated with higher platelet counts and unique clinical presentations that differ from typical CML cases [216,217]. Moreover, rare fusions like e6a2 have been linked to an aggressive disease course and poor prognosis, often requiring more intensive treatment strategies [210].

Additionally, NGS provides high-resolution detection of *BCR::ABL1* kinase domain mutations (Table 8), which are a major cause of resistance to TKIs. While first-line therapy with imatinib is effective in many patients, approximately 30% develop resistance, often due to mutations in the *BCR::ABL1* kinase domain [218]. Specific mutations, such as *T315I*, are known to confer resistance to imatinib and second-generation TKIs like dasatinib and nilotinib. The early detection of such mutations through NGS allows for the strategic selection of alternative inhibitors, such as ponatinib, which retains activity against *T315I* [219]. Other common mutations include *Y253H*, *E255K/V*, and *F359V*, which are associated with varying degrees of resistance to imatinib, dasatinib, and nilotinib [220]. The localization of these mutations within the kinase domain is critical, as mutations in the P-loop region, such as *G250E* and *E255K*, tend to confer high resistance and are associated with worse clinical outcomes [221]. Mutations can emerge under the selective pressure of TKI therapy, leading to clonal evolution and disease progression [222]. Recent advances in targeted therapy, including the development of allosteric inhibitors such as asciminib, offer new therapeutic options for patients with resistant mutations [223]. By identifying low-frequency resistant mutations before clinical relapse, NGS allows for earlier therapeutic intervention and personalized treatment modifications [224].

Moreover, NGS has identified compound mutations, where multiple resistance-associated mutations co-exist within the same leukemic clone, leading to complex drug resistance patterns [92]. Studies have shown that compound mutations can emerge through sequential, branching, or parallel routes under selective pressure from TKI therapy, resulting in complex clonal architectures [225]. While third-generation TKIs like ponatinib are effective against most single *BCR::ABL1* mutations, certain compound mutations, such as *T315I/E255K* and *T315I/F359V*, remain highly resistant even to ponatinib, limiting treatment options [208]. The detection of compound mutations is essential for optimal therapy selection, yet conventional sequencing methods often fail to distinguish between polyclonal mutations (present in separate clones) and true compound mutations (within the same clone) [92]. Advanced techniques such as ddPCR and NGS have improved the ability to accurately identify compound mutations and guide therapeutic decisions [226]. The high frequency of compound mutations in TKI-resistant CML underscores the need for routine molecular monitoring and the development of novel therapeutic approaches, including allosteric inhibitors and combination treatment strategies [227]. Overall, NGS’s ability to sequence entire mutational landscapes has refined therapeutic strategies by guiding treatment escalation, de-escalation, or combination approaches tailored to each patient’s mutational profile [228].

Beyond initial diagnosis and therapy selection, NGS has been instrumental in monitoring disease progression and treatment response in CML. The persistence or re-emergence of *BCR::ABL1* transcripts at low levels following treatment is a strong predictor of relapse, and conventional qPCR lacks the sensitivity to detect minor subclones harboring resistance mutations [229]. NGS-based MRD monitoring provides ultra-sensitive detection of leukemic clones, enabling earlier intervention before clinical progression occurs. Studies have shown that NGS can detect emerging TKI-resistant clones months before they become dominant, allowing for proactive treatment adjustments [205]. Another study demonstrated that patients who achieved deep molecular remission (DMR) but still harbored detectable *BCR::ABL1* DNA via NGS were more likely to relapse after TKI cessation [230]. It highlights the clinical importance of NGS in MRD monitoring for CML, particularly in patients attempting treatment-free remission (TFR) after TKI therapy. Traditionally, molecular response in CML is assessed using qPCR to measure *BCR::ABL1* transcript levels (Table 9). Patients who achieve a DMR, defined as *BCR::ABL1* levels below 0.01% (MR4) or undetectable by qPCR (MR4.5 or MR5), are considered potential candidates for TKI discontinuation [231,232]. However, qPCR has a detection limit, and some patients who appear to be in DMR may still harbor leukemic clones at a molecular level that qPCR cannot detect. NGS provides an alternative approach by detecting *BCR::ABL1* DNA at very low levels, sometimes beyond the limits of PCR [233,234].

Additionally, NGS has been used to uncover secondary genomic alterations beyond *BCR::ABL1*, such as mutations in *SETBP1*, *TP53*, and *ASXL1* (Table 8), which contribute to disease acceleration and blast crisis transformation [235]. For instance, *SETBP1* mutations in CML, particularly in its atypical form (aCML), have been identified as recurrent genetic alterations associated with poor prognosis and aggressive disease progression. These mutations occur in approximately 24% of aCML cases and are frequently located within codons 858–871, leading to a gain-of-function effect that disrupts ubiquitination, thereby increasing *SETBP1* protein levels and promoting leukemic cell proliferation [236,237]. Functionally, *SETBP1* mutations result in the inhibition of the tumor suppressor PP2A, leading to enhanced leukemic cell survival and resistance to apoptosis [238]. Clinically, patients with *SETBP1* mutations tend to present with higher white blood cell counts and an increased risk of transformation to AML, making this mutation an important prognostic marker [239]. In addition, *SETBP1* mutations often co-occur with other high-risk genetic abnormalities, such as *-7/del(7q)*, further contributing to adverse clinical outcomes [240]. Given their significant impact on disease biology, *SETBP1* mutations are now considered a key molecular marker in aCML, and their detection may aid in refining risk stratification and guiding more aggressive treatment approaches, including early consideration of allogeneic stem cell transplantation. Meanwhile, *TP53* mutations in CML are relatively rare in the chronic phase but become more prevalent in advanced stages, particularly during blast crisis [241]. These mutations are strongly associated with disease progression, therapy resistance, and poor prognosis. Studies have shown that *TP53* alterations, including mutations and deletions on chromosome 17p, occur in up to 30% of blast crisis cases, often leading to treatment failure with TKIs [183,242]. Unlike *BCR::ABL1* kinase domain mutations, which are the primary mechanism of TKI resistance, *TP53* mutations drive genomic instability and promote leukemic transformation. Some cases also exhibit a correlation between *TP53* deletions and complex chromosomal rearrangements, further worsening prognosis [243]. Given their role in disease progression, *TP53* mutations are considered a marker of clonal evolution and may indicate the need for alternative treatment strategies, such as allogeneic stem cell transplantation or novel targeted therapies. Conversely, *TP53* mutations in CLL are more common and occur in approximately 10–20% of cases, with their frequency increasing in relapsed or refractory disease [244]. Unlike in CML, where *TP53* mutations are linked to disease progression to blast crisis, in CLL, they are associated with poor response to chemoimmunotherapy, particularly to purine analogs such as fludarabine. While *TP53* mutations in both CML and CLL confer poor prognosis, their implications differ; in CML, they mark transformation to a more advanced disease stage, whereas in CLL, they drive resistance to standard therapies and necessitate targeted treatment approaches [245]. Next, *AXL* mutations in CML are relatively rare, but overexpression of *AXL*, a receptor tyrosine kinase, has been strongly associated with disease progression and resistance to TKIs [246]. *AXL* is part of the TAM (Tyro3, AXL, Mer) family of kinases, which plays a role in cell survival, proliferation, and immune evasion. Studies have shown that *AXL* is upregulated in CML, particularly in cases resistant to imatinib, suggesting a role in acquired resistance mechanisms [246,247]. The overexpression of *AXL* has been linked to activation of downstream pathways such as PI3K/AKT and STAT5, which contribute to leukemic cell survival and drug resistance. Additionally, *AXL* appears to regulate the persistence of leukemia stem and progenitor cells, independent of *BCR::ABL1* activity, making it a potential therapeutic target even in TKI-resistant cases [248]. Pharmacological inhibition of *AXL* using BGB324 has demonstrated efficacy in overcoming both TKI-sensitive and TKI-resistant CML cells, including those harboring the *T315I* mutation, which is resistant to most TKIs except ponatinib [248]. Furthermore, targeting *AXL* has been shown to decrease leukemia cell proliferation and improve responses to combination therapies, particularly in cases with persistent minimal residual disease [249].

The RAS/MAPK signaling pathway plays a significant role in the pathogenesis of CML, particularly in promoting leukemic cell survival, proliferation, and resistance to apoptosis. This pathway is activated downstream of the BCR::ABL1 fusion protein, which is the hallmark of CML. The BCR::ABL1 oncoprotein constitutively activates multiple signaling pathways, including RAS/MAPK, PI3K/AKT, and JAK/STAT, which collectively drive the uncontrolled growth of leukemic cells [250,251]. The RAS/MAPK cascade is initiated when BCR::ABL1 activates RAS, which subsequently triggers RAF kinases, leading to the phosphorylation of MEK1/2 and ERK1/2. This signaling cascade enhances cell proliferation and inhibits apoptosis, thereby contributing to CML progression. Studies have shown that the activation of this pathway is linked to the resistance of leukemic stem cells to TKIs, such as imatinib, dasatinib, and nilotinib [252]. Additionally, blocking the RAS/MAPK pathway with specific inhibitors such as MEK inhibitors has demonstrated potential in reducing CML cell proliferation and enhancing sensitivity to TKIs [253]. While RAS mutations are rare in chronic-phase CML, they have been reported more frequently in the blast crisis phase, where they contribute to disease progression and drug resistance [254]. The increased activation of the RAS/MAPK pathway in advanced CML suggests that this signaling axis plays a crucial role in leukemic transformation. *NRAS* and *KRAS* mutations are relatively rare in the chronic phase but become more prevalent in advanced disease stages, particularly during blast crisis. These mutations play a role in leukemic transformation by driving uncontrolled cell proliferation and survival through constitutive activation of the RAS/MAPK signaling pathway [255,256]. Studies have shown that *NRAS* mutations are more frequent than *KRAS* mutations in myeloid malignancies, with *HRAS* mutations being exceedingly rare. Mutations at codons 12, 13, or 61 of *NRAS* and *KRAS* result in a constitutively active GTP-bound state, leading to persistent downstream signaling through RAF-MEK-ERK and PI3K-AKT pathways [257]. This contributes to resistance against apoptosis and enhances leukemic stem cell self-renewal, which may explain why these mutations are more frequently found in patients with blast crisis rather than in the chronic phase. While these mutations are not commonly screened for in early-stage CML, their detection in advanced disease may provide important prognostic insights and influence therapeutic strategies. Overall, these findings support the emerging role of NGS in capturing the full spectrum of genetic evolution in CML, offering a more comprehensive approach to long-term disease management [258].

## 8. When to Perform NGS in Leukemia Disease Course?

The frequency of NGS testing in leukemia management is still under investigation, with recommendations varying based on disease type, risk stratification, and treatment phase [208]. Current guidelines suggest that NGS should be performed at diagnosis to identify clinically relevant mutations that guide prognosis and treatment decisions, particularly in acute leukemias such as AML and ALL [88]. At diagnosis, targeted NGS panels help classify patients into appropriate risk groups based on mutations in genes such as *FLT3*, *NPM1*, *TP53*, and *IDH1/IDH2*, enabling personalized therapy selection. Meanwhile, at CML diagnosis, NGS is recommended to detect both *BCR::ABL1* fusion transcripts and additional mutations that may impact prognosis and treatment selection. While qPCR is the gold standard for detecting *BCR::ABL1*, NGS allows for a more comprehensive genetic profile, identifying co-occurring mutations in genes like *ASXL1*, *RUNX1*, and *TP53*, which have been associated with worse outcomes and potential resistance to TKIs [92]. Following diagnosis, the timing of NGS testing varies based on disease course and treatment milestones. For AML, repeat NGS is often recommended at relapse to identify emerging resistance mutations or clonal evolution, which may necessitate changes in therapy, such as switching to targeted inhibitors or considering allogeneic stem cell transplantation [259]. Additionally, in chronic leukemias such as CLL, NGS is useful at treatment initiation and disease progression, particularly for identifying *TP53* mutations, which predict poor response to chemoimmunotherapy and favor the use of targeted agents like BTK inhibitors [182,183]. In CML, NGS is also recommended when there is an increase in *BCR::ABL1* transcript levels during treatment. Rising transcript levels can indicate emerging resistance, and NGS helps identify resistance mutations within the BCR::ABL1 kinase domain before they become clinically significant [92,208]. Identifying mutations like *T315I* or compound mutations allows clinicians to switch to appropriate second- or third-generation TKIs before complete treatment failure.

In MRD monitoring, NGS is being explored for ultra-sensitive detection of low-level leukemia cells, particularly post-treatment in AML and ALL. Studies suggest that patients with persistent leukemia-associated mutations detected by NGS, even in the absence of morphological relapse, are at higher risk of recurrence [94]. While qPCR remains the standard for MRD assessment, NGS may be used at key post-treatment time points, such as after induction chemotherapy, before consolidation, and before TFR attempts in CML. Before TKI discontinuation in CML, NGS can play a role in assessing MRD at an ultra-sensitive level. While standard qPCR assesses *BCR::ABL1* transcript levels, ultra-deep NGS can detect residual leukemic clones that may predict relapse. Studies have shown that patients who achieve DMR but still harbor detectable *BCR::ABL1* DNA by NGS have a higher risk of relapse after stopping TKIs [229]. NGS is also valuable for monitoring patients who have discontinued TKIs due to sustained deep remission. While qPCR is typically used for routine MRD monitoring, periodic NGS testing can help detect very low-level residual clones that could signal impending relapse [259]. Finally, if CML progresses to the accelerated or blast phase, NGS becomes essential for detecting secondary mutations beyond *BCR::ABL1*. Disease progression is often associated with the accumulation of additional genetic alterations, including mutations in *TP53*, *RUNX1*, and *SETBP1*, which contribute to TKI resistance and poor prognosis [260]. At this stage, NGS guides treatment strategies, including switching TKIs or considering allogeneic stem cell transplantation. Despite these insights, there is no universal agreement on a fixed frequency for NGS testing, as its role depends on leukemia type, disease stage, and available targeted therapies.

## 9. Challenges and Future Perspectives

### 9.1. Standardization and Interpretation Challenges

One of the primary challenges in the clinical implementation of NGS in leukemia diagnostics is the lack of standardization in sequencing protocols, data processing, and variant interpretation. Unlike conventional diagnostic methods such as karyotyping and FISH, which have well-established guidelines, NGS technologies vary significantly between laboratories in terms of sequencing platforms, target gene panels, and bioinformatics pipelines. The absence of universal standards complicates the reproducibility of results, making it difficult to compare findings across different institutions [261]. Efforts to establish standardized quality control measures, such as minimum sequencing depth and uniform variant reporting criteria, are crucial for ensuring consistency in NGS-based leukemia diagnostics [91]. The interpretation of NGS data presents another significant hurdle, as the vast number of genetic variants detected often includes mutations of uncertain significance (VUS) [262]. In leukemia, distinguishing between driver mutations, which contribute to disease pathogenesis, and passenger mutations, which have no functional impact, is essential for clinical decision-making. However, many variants identified by NGS have not been well-characterized in existing databases, leading to ambiguity in their clinical relevance. The use of curated genomic databases such as ClinVar and COSMIC, along with functional validation studies, is necessary to refine variant classification [120,263]. Additionally, the integration of artificial intelligence and machine learning models is being explored as a means to automate and enhance variant interpretation, reducing the burden on clinicians and bioinformaticians [264,265].

Regulatory oversight and clinical validation further complicate the widespread adoption of NGS in leukemia diagnostics. The U.S. Food and Drug Administration (FDA) and other regulatory agencies have recognized the need for establishing guidelines to ensure the clinical reliability of NGS-based testing. The FDA’s regulatory approach aims to balance scientific innovation with the need for standardized oversight to protect patient safety. In 2018, the FDA finalized its guidance on NGS-based in vitro diagnostic tests, providing a framework for analytical validation and performance criteria, particularly for identifying genetic variants relevant to leukemia and other diseases [266]. This guidance helps laboratories and test developers establish regulatory-compliant NGS assays for clinical use. Additionally, the FDA has outlined criteria for evaluating the clinical validity of NGS-based tests, incorporating data from recognized genetic variant databases to support the interpretation of genomic findings. One of the key milestones in the FDA’s regulatory efforts was the approval of comprehensive NGS panels for detecting actionable genomic aberrations in cancer, including leukemia. These approvals established a regulatory pathway for NGS-based companion diagnostics, ensuring that these tests meet stringent accuracy and reproducibility standards before being integrated into clinical practice [267]. However, there have been ongoing debates regarding the extent of FDA oversight, particularly concerning laboratory-developed tests (LDTs), which make up a large portion of NGS-based leukemia diagnostics. The FDA’s regulatory approach has been scrutinized for potentially increasing the cost and time required for test development while also ensuring that assays used in clinical decision-making are reliable and reproducible [268]. The FDA has also collaborated with the Centers for Medicare and Medicaid Services (CMS) to align regulatory standards with reimbursement policies, as seen in the decision to grant Medicare coverage for FDA-approved NGS tests used in cancer treatment [269]. This alignment has facilitated broader access to NGS testing for leukemia patients, allowing for more precise molecular profiling and personalized treatment approaches.

The regulation of NGS in leukemia management in Europe is guided by a combination of European Union (EU) directives, national regulations, and recommendations from organizations such as the ELN. The implementation of NGS in clinical practice varies widely across EU countries, with disparities in regulatory policies and reimbursement structures affecting patient access. The European In Vitro Diagnostic Medical Devices Regulation (IVDR), which came into effect in 2022, established stricter requirements for NGS-based diagnostic tests, ensuring they meet high standards of analytical validity and clinical performance. However, despite these regulatory advances, the adoption of NGS in routine leukemia management remains inconsistent due to differences in national guideline implementations and reimbursement policies [270]. The ELN has played a crucial role in integrating NGS into leukemia diagnosis and treatment guidelines. ELN recommendations emphasize the importance of NGS in identifying key mutations for risk stratification and guiding personalized therapies, particularly in AML and CLL [271,272]. However, regulatory frameworks are still evolving, and there is a significant challenge in the widespread implementation of NGS due to the lack of standardized protocols across different countries, leading to variability in how NGS data are interpreted and used in clinical decision-making. Moreover, there is no universally accepted approach for validating NGS assays. Unlike single-gene tests, which undergo rigorous analytical validation, NGS panels cover multiple genes simultaneously, increasing the complexity of quality assurance. Establishing proficiency testing programs and cross-laboratory validation studies will be essential in addressing these concerns and ensuring that NGS results are both accurate and actionable [273]. Future advancements in NGS standardization and interpretation will likely involve greater collaboration between research institutions, regulatory bodies, and clinical laboratories. The development of international guidelines for sequencing quality control, variant classification, and data reporting will facilitate the seamless integration of NGS into leukemia management. Another barrier to NGS adoption in Europe is the uneven reimbursement landscape, which affects patient access to these advanced genomic tests. While some countries, such as Germany and France, have integrated NGS into their national healthcare systems with established reimbursement mechanisms, other EU nations still struggle with financial and logistical barriers that limit NGS availability for leukemia patients [274]. Policymakers and healthcare stakeholders have called for a more harmonized approach to NGS regulation and reimbursement, advocating for multi-stakeholder collaborations to streamline NGS implementation across Europe.

### 9.2. Other Barriers to Widespread Clinical Application

Beyond regulatory and interpretation challenges, several other barriers hinder the widespread application of NGS in clinical leukemia management. One major limitation is the high cost of NGS testing, which remains a significant financial burden for both healthcare institutions and patients. While the cost of sequencing has decreased over the years, comprehensive leukemia panels, particularly WGS or WES, remain expensive, making them inaccessible in many healthcare settings. A survey of U.S. oncologists and hematologists revealed that limited reimbursement from insurance providers is a primary concern, leading to financial constraints that prevent the routine use of NGS in leukemia diagnostics [275]. Without consistent coverage policies, hospitals and laboratories face difficulties in integrating NGS into standard leukemia care. Another major challenge is the turnaround time for NGS results, which can take days to weeks, depending on the sequencing approach. In acute leukemias, where rapid treatment decisions are crucial, delays in obtaining NGS data may render the information less useful for immediate clinical decision-making [276]. While advances in automation and bioinformatics are improving sequencing efficiency, many institutions still struggle with optimizing workflows to reduce turnaround time [277]. Additionally, the requirement for highly skilled personnel to perform sequencing and analyze complex genomic data remains a barrier, particularly in smaller hospitals and laboratories without dedicated molecular oncology teams. The lack of standardized guidelines for incorporating NGS into leukemia management also contributes to its limited adoption. While organizations like the ELN and the National Comprehensive Cancer Network (NCCN) have provided recommendations on using NGS for risk stratification, there is no universal consensus on when and how often NGS should be performed in leukemia patients [208,271,278]. This uncertainty has led to variations in clinical practice, where some physicians rely on traditional molecular techniques like PCR and FISH rather than adopting NGS-based approaches [89]. Additionally, evidence gaps in clinical utility remain a significant challenge. While NGS has expanded our understanding of leukemia genomics, not all detected mutations have immediate therapeutic implications [279]. Many physicians hesitate to order NGS tests because of concerns about the actionable value of certain mutations, particularly in cases where targeted therapies are not yet available. A survey found that 80.1% of physicians identified the lack of strong evidence linking NGS findings to treatment decisions as a major barrier to routine use [280]. Expanding clinical trials and real-world studies to validate the prognostic and therapeutic significance of emerging leukemia-associated mutations could help bridge this gap.

### 9.3. Integration of NGS with Other Omics Technologies

The integration of NGS with other omics technologies, such as transcriptomics, proteomics, and metabolomics, is transforming leukemia research by providing a comprehensive understanding of disease biology [102,281]. While NGS primarily focuses on genomic alterations, integrating multiple omics layers allows researchers to analyze gene expression patterns, protein interactions, and metabolic changes that drive leukemia progression. Transcriptomic profiling through RNA-Seq, for instance, helps identify aberrant gene expression signatures and oncogenic fusion transcripts that may not be detected by DNA sequencing alone [282]. This approach has been instrumental in refining leukemia subtypes and uncovering novel therapeutic targets [283]. Additionally, single-cell transcriptomics has provided deeper insights into clonal heterogeneity, enabling the identification of rare leukemia stem cell populations that contribute to relapse and treatment resistance [284]. Proteomics and metabolomics further enhance leukemia characterization by elucidating functional alterations at the protein and metabolic levels. Proteomic studies have identified post-translational modifications that regulate leukemia cell signaling pathways, offering potential drug targets beyond genetic mutations [285]. Mass spectrometry-based proteomics has revealed dysregulated kinase activity in AML, leading to the development of kinase inhibitors tailored to specific protein alterations [286]. Similarly, metabolomics has provided insights into metabolic reprogramming in leukemia, demonstrating that leukemic cells rely on altered energy metabolism for survival and proliferation [287]. The integration of metabolomics with NGS data has been particularly useful in understanding how metabolic vulnerabilities can be exploited for therapeutic intervention, such as targeting amino acid metabolism in leukemia cells [288].

Multi-omics integration also plays a crucial role in overcoming the limitations of NGS in predicting drug responses and treatment resistance. By combining genomics with epigenomics, researchers have identified DNA methylation patterns and histone modifications that influence leukemia progression and therapy resistance [289]. For example, epigenetic alterations in *DNMT3A* and *TET2* have been shown to impact response to hypomethylating agents in AML, highlighting the importance of epigenomic profiling in therapeutic decision-making [290,291]. Additionally, multi-omics approaches have been employed to develop predictive models for leukemia treatment outcomes, incorporating genomic, transcriptomic, and proteomic data to refine risk stratification and personalize therapy [292,293]. Despite its potential, integrating multi-omics data presents significant challenges, including the complexity of data interpretation, the need for standardized analytical frameworks, and the high computational demands of processing large-scale datasets [294]. Advanced bioinformatics tools and machine learning algorithms are being developed to address these challenges, enabling more effective integration of diverse omics datasets [295,296]. The future of leukemia research lies in leveraging multi-omics approaches to achieve a system-level understanding of the disease, paving the way for more precise diagnostics and innovative therapeutic strategies.

### 9.4. Potential for Real-Time NGS in Clinical Decision-Making

The potential for real-time NGS in clinical decision-making is a rapidly evolving area that could revolutionize leukemia management by providing near-instant genomic insights for treatment selection. Traditional sequencing methods often require days to weeks to process results, delaying critical treatment decisions. However, real-time NGS approaches, particularly with nanopore sequencing technologies, have demonstrated the ability to classify leukemias in minutes, making it feasible to incorporate genomic data into immediate therapeutic strategies [297]. In a recent study, real-time transcriptomic profiling using nanopore sequencing successfully distinguished molecular subtypes of ALL within minutes, highlighting the potential for rapid leukemia diagnosis and classification [24]. One of the most promising applications of real-time NGS is in guiding treatment decisions for patients with relapsed or refractory leukemia. The ability to quickly detect mutations associated with drug resistance, such as *FLT3* or *TP53* mutations in AML, allows clinicians to adjust therapy before treatment failure occurs. Studies have shown that comprehensive NGS panels can provide actionable genomic data that influence treatment selection, such as identifying patients who may benefit from targeted inhibitors like FLT3 or IDH1/2 inhibitors [298]. Additionally, the integration of real-time NGS into clinical workflows has been proposed as a strategy to refine treatment sequencing patterns in CLL, enabling a more dynamic and personalized approach to therapy selection [299].

Beyond initial diagnosis and therapy selection, real-time NGS is expected to play a key role in monitoring MRD and predicting relapse. Current MRD detection methods, such as flow cytometry and PCR, have limitations in sensitivity and may not capture emerging resistant clones. Real-time NGS offers ultra-sensitive monitoring of leukemia cells, allowing clinicians to detect molecular relapse earlier than conventional methods. A comparative study demonstrated that NGS-based MRD monitoring provided superior sensitivity compared to flow cytometry, underscoring its potential to guide early treatment modifications [89,300]. As sequencing technologies become more efficient and cost-effective, the implementation of real-time NGS for routine leukemia monitoring is expected to enhance patient outcomes by enabling timely therapeutic interventions [102]. Despite its advantages, real-time NGS faces challenges related to data interpretation, cost, and infrastructure requirements. The volume of sequencing data generated in real time necessitates advanced bioinformatics pipelines capable of rapid analysis and clinical annotation [301]. Moreover, integrating real-time sequencing into hospital settings requires significant investment in sequencing platforms and trained personnel. However, ongoing developments in machine learning-driven variant interpretation and cloud-based genomic analysis platforms are expected to mitigate these barriers, making real-time NGS increasingly accessible for clinical use [302]. As these technologies continue to mature, real-time NGS is poised to become a cornerstone of precision medicine, transforming leukemia diagnostics and treatment decision-making.

### 9.5. Drug Development and Precision Medicine

NGS has become an essential tool in drug development and precision medicine by identifying genetic alterations that can be targeted with novel therapeutics. Traditional drug development relies on broad-spectrum cytotoxic agents, but NGS has shifted this paradigm toward the design of molecularly targeted therapies tailored to specific genetic mutations. In leukemia, this has led to the approval of inhibitors such as FLT3 inhibitors for *FLT3*-mutant AML and BCL2 inhibitors for CLL. By analyzing the genomic landscape of leukemias, NGS enables the identification of novel drug targets, allowing pharmaceutical companies to develop precision medicines aimed at specific molecular pathways [303]. The application of NGS in genetically stratified clinical trials has revolutionized oncology drug testing, making trials more efficient and outcome-driven. Instead of treating all patients with a one-size-fits-all approach, NGS allows for the enrollment of participants based on their specific mutational profiles, ensuring that only those who are most likely to benefit from a particular therapy receive it. This approach increases the success rates of clinical trials and reduces the exposure of patients to ineffective treatments. For example, the use of NGS-guided patient selection in clinical trials for IDH1 and IDH2 inhibitors in AML has demonstrated improved response rates compared to conventional trial designs [304]. Additionally, real-time genomic monitoring through NGS enables adaptive clinical trial designs, where treatment regimens can be modified in response to emerging resistance mutations [305].

**Figure 4:**
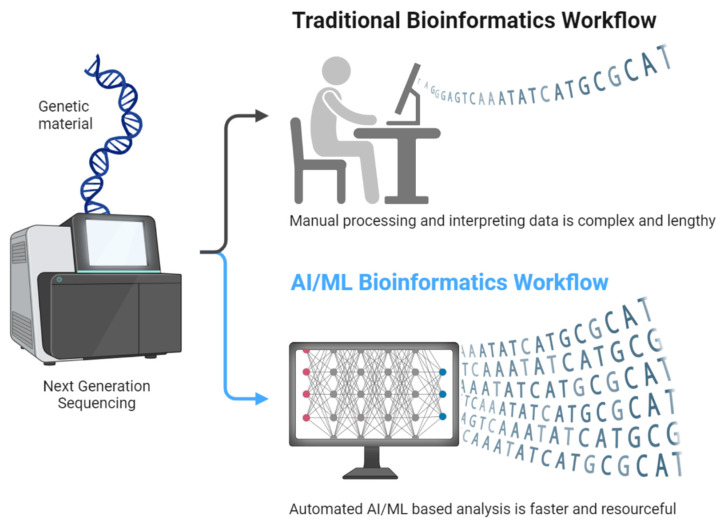
Potential Application of Artificial Intelligence, Including Machine Learning to Process NGS Data.

Beyond individual targeted therapies, NGS plays a crucial role in combination drug development, where multiple agents are tested together to overcome resistance mechanisms. Multi-omics data from NGS, transcriptomics, and proteomics have identified synergistic drug combinations that enhance treatment efficacy. For instance, studies integrating NGS and RNA sequencing have revealed that combining BCL2 inhibitors with hypomethylating agents in AML results in greater leukemia cell death than either agent alone. These findings have led to the approval of venetoclax in combination with azacitidine, a treatment now widely used for elderly AML patients who are ineligible for intensive chemotherapy [306]. Despite these advances, significant challenges remain in translating NGS findings into clinically approved therapies. One major barrier is the complexity of interpreting the vast amount of genomic data generated, as not all detected mutations are actionable or have a well-defined therapeutic intervention. Additionally, regulatory agencies such as the U.S. FDA require rigorous validation of NGS-based biomarkers before they can be integrated into drug approval processes. Overcoming these challenges will require further collaboration between researchers, clinicians, and regulatory bodies to standardize genomic data interpretation and integrate NGS findings into routine clinical practice [307]. As precision medicine continues to evolve, the role of NGS in drug discovery and development will expand, leading to more personalized and effective leukemia treatments. The integration of artificial intelligence and machine learning into NGS analysis (Figure 4) is expected to further streamline drug target identification, improving the efficiency of therapeutic development. With ongoing advancements in sequencing technologies, the future of leukemia treatment will increasingly rely on genomics-driven approaches that ensure the right drug is given to the right patient at the right time.

## 10. Conclusions

**Figure 5:**
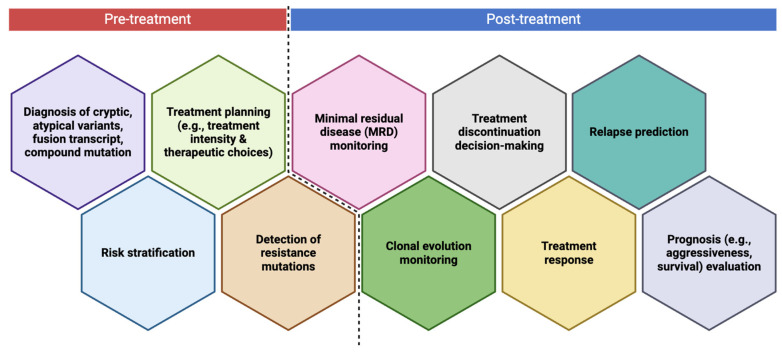
The Role of Next-Generation Sequencing in Leukemia Management. In the pre-treatment phase, NGS assists in diagnosing cryptic and atypical variants, risk stratification, treatment planning, and detection of resistance mutations. In the post-treatment phase, NGS is utilized for minimal residual disease (MRD) monitoring, clonal evolution monitoring, treatment response assessment, relapse prediction, treatment discontinuation decisions, and prognosis evaluation. These insights contribute to personalized leukemia management and improved patient outcomes.

Next-generation sequencing has revolutionized the diagnosis, prognosis, and treatment of leukemia (Figure 5) by enabling comprehensive genomic profiling with unprecedented accuracy. Compared to conventional diagnostic methods, NGS provides deeper insights into genetic alterations, clonal evolution, and resistance mechanisms, facilitating personalized treatment strategies. While challenges such as data interpretation, standardization, and cost remain, continuous advancements in sequencing technologies and bioinformatics are improving their clinical applicability. As precision medicine evolves, NGS is expected to become an integral tool in leukemia management, driving innovations in targeted therapy and real-time disease monitoring. Future research should focus on optimizing its integration into routine clinical practice, ensuring broader accessibility, and refining its predictive capabilities for improved patient outcomes.

## Figures and Tables

**Table 1:** Comparison of ALL, AML, CLL, and CML.

Feature	Acute Lymphoblastic Leukemia (ALL)	Acute Myeloid Leukemia (AML)	Chronic Lymphocytic Leukemia (CLL)	Chronic Myeloid Leukemia (CML)
Cell of Origin	Lymphoid progenitor cells (B or T cells)	Myeloid progenitor cells	Mature B lymphocytes	Myeloid stem cells
Age Group Affected	Most common in children, but also occurs in adults	More common in adults, especially older than 60 years	Primarily affects older adults (>60 years)	Typically affects middle-aged adults (40–60 years)
Onset and Progression	Rapid onset and aggressive course	Rapid onset and aggressive course	Slow progression, often asymptomatic initially	Gradual progression, can transition to a blast crisis
Common Genetic Abnormalities	*ETV6::RUNX1*, *IKZF1* deletions, *CRLF2* rearrangements	*FLT3*, *NPM1*, *TP53, IDH1/2*, *RUNX1* mutations	*TP53*, *NOTCH1*, *SF3B1* mutations, *del(13q14)*, trisomy 12	*BCR::ABL1* fusion (Philadelphia chromosome)
Symptoms	Fatigue, fever, bone pain, lymphadenopathy, bleeding	Fatigue, anemia, recurrent infections, bleeding	Lymphadenopathy, fatigue, hepatosplenomegaly	Fatigue, weight loss, splenomegaly
Diagnostic Methods	Bone marrow biopsy, flow cytometry, cytogenetics, PCR, NGS	Bone marrow biopsy, flow cytometry, cytogenetics, PCR, NGS	Peripheral blood smear, flow cytometry, FISH, NGS	PCR for *BCR::ABL1*, FISH, cytogenetics, NGS
Treatment Approaches	Chemotherapy, targeted therapy (TKIs for Ph+ ALL), immunotherapy, bone marrow transplant	Chemotherapy, targeted therapy (FLT3 inhibitors, IDH inhibitors), bone marrow transplant	BTK inhibitors (ibrutinib, acalabrutinib), BCL2 inhibitors (venetoclax), monoclonal antibodies (rituximab)	Tyrosine kinase inhibitors (TKIs) such as imatinib, nilotinib, and dasatinib
Prognosis	Highly variable; better in children, worse in adults	Prognosis depends on mutations and risk group; high-risk mutations have a poor prognosis	Generally good with targeted therapies, but incurable	Excellent long-term prognosis with TKIs; risk of transformation to blast crisis

**Table 2:** Comparison of Diagnostic Methods in Acute and Chronic Leukemia.

Diagnostic Method	Acute Lymphoblastic Leukemia (ALL)	Acute Myeloid Leukemia (AML)	Chronic Lymphocytic Leukemia (CLL)	Chronic Myeloid Leukemia (CML)
Peripheral Blood Smear	Increased lymphoblasts, anemia, thrombocytopenia	Increased myeloblasts, Auer rods present	Increased mature lymphocytes, smudge cells	Increased myeloid precursors, left-shifted granulocytes
Bone Marrow Aspiration and Biopsy	Hypercellular marrow with >20% lymphoblasts	Hypercellular marrow with >20% myeloblasts	Not always required, but shows lymphocytic infiltration	Hypercellular marrow with granulocytic hyperplasia
Flow Cytometry	Identifies B-cell (CD19, CD22) or T-cell (CD3, CD7) markers	Identifies myeloid markers (CD13, CD33, CD117)	Confirms monoclonal B-cell population (CD5, CD19, CD23)	Not routinely needed, but can confirm granulocytic lineage
Cytogenetics and Karyotyping	Detects chromosomal translocations (*ETV6::RUNX1*, *BCR::ABL1*)	Identifies abnormalities like *t(8;21)*, *inv(16)*, or complex karyotypes	Detects chromosomal abnormalities like *del(13q)*, trisomy 12	Detects Philadelphia chromosome (*t(9;22)*)
Fluorescence In Situ Hybridization (FISH)	Confirms *BCR::ABL1*, *MLL* rearrangements	Detects *RUNX1-RUNX1T1*, *PML::RARA* fusions	Identifies *TP53* deletion, *ATM* deletion	Detects *BCR::ABL1* fusion gene in non-dividing cells using fluorescent probes.
Polymerase Chain Reaction (PCR)	Detects *BCR::ABL1*, *ETV6::RUNX1*, *IKZF1* deletions	Identifies *FLT3*, *NPM1*, *IDH1/2* mutations	Detects *IGHV* mutation, *NOTCH1* mutations	Quantifies *BCR::ABL1* transcript levels for monitoring minimal residual disease.
Next-Generation Sequencing (NGS)	Identifies risk-associated mutations (*CRLF2*, *PAX5*, *IKZF1*)	Detects multiple mutations for risk stratification (*FLT3*, *TP53*, *DNMT3A*)	Provides full mutational landscape (*TP53*, *NOTCH1*, *SF3B1*)	Comprehensively sequences genes to detect mutations, fusions, and clonal evolution.
Minimal Residual Disease (MRD) Monitoring *	Flow cytometry (10^−4^–10^−5^), PCR (10^−5^–10^−6^), NGS (10^−6^, targeted or error-corrected ultra-deep sequencing)	Flow cytometry (10^−3^–10^−4^), PCR (10^−4^–10^−5^), NGS (10^−5^–10^−6^, targeted deep sequencing)	PCR (10^−4^–10^−5^), NGS (10^−5^–10^−6^, error-corrected ultra-deep sequencing)	qPCR (10^−5^), NGS (10^−5^–10^−6^, targeted deep sequencing for *BCR::ABL1* transcript monitoring)

*: The sensitivity values for MRD monitoring indicate the lowest detectable leukemia cell fraction. Flow cytometry typically detects 1 in 10,000 (10^−4^) to 1 in 100,000 (10^−5^) cells, while PCR improves sensitivity from 1 in 100,000 (10^−5^) to 1 in 1,000,000 (10^−6^). NGS provides the highest sensitivity, with targeted deep sequencing offering detection at 10^−5^ to 10^−6^, while error-corrected ultra-deep sequencing (such as duplex sequencing) can reach sensitivities of 10^−6^ or lower. This level of precision is particularly useful in tracking subclonal mutations and detecting emerging resistance in leukemia patients.

**Table 3:** Comparison of Different Types of Next-Generation Sequencing (NGS) Approaches.

NGS Type	Description	Coverage	Sensitivity	Advantages	Limitations	Common Applications in Leukemia
Whole-Genome Sequencing (WGS)	Sequences the entire genome, including coding and non-coding regions	Comprehensive	Moderate (10^−3^–10^−5^)	Detects all genetic variations, including structural variants and non-coding mutations	High cost, large data volume, complex interpretation	Research, discovery of novel mutations, understanding clonal evolution
Whole-Exome Sequencing (WES)	Sequences only the protein-coding regions (exome), which comprise ~1–2% of the genome	Protein-coding regions	High (10^−4^–10^−5^)	Cost-effective compared to WGS, detects clinically relevant mutations	Misses non-coding variants and structural abnormalities	Identifying actionable mutations in leukemia, risk stratification
Targeted Gene Panel Sequencing	Focuses on a preselected set of leukemia-associated genes	Limited to selected genes	Very high (10^−5^–10^−6^)	Cost-effective, high-depth, detects low-frequency mutations	Limited to known genes, cannot detect novel mutations	Routine clinical diagnostics, mutation-driven therapy selection, minimal residual disease (MRD) monitoring
RNA Sequencing (RNA-Seq)	Analyzes the transcriptome to measure gene expression and detect fusion genes	Coding and non-coding RNA	Moderate (10^−3^–10^−5^)	Detects gene fusions, alternative splicing, and expression changes	Requires high-quality RNA, can be influenced by degradation	Identifying leukemia-specific fusion genes (*BCR::ABL1*, *ETV6::RUNX1*), transcriptomic profiling
Single-Cell Sequencing (scRNA-Seq)	Analyzes genetic information from individual cells instead of bulk tissue	Single-cell resolution	High (10^−4^–10^−5^)	Identifies rare subclones, provides insight into tumor heterogeneity	Expensive, complex data interpretation requires specialized bioinformatics	Studying clonal evolution, therapy resistance, leukemia stem cell characterization
Error-Corrected Ultra-Deep Sequencing (Duplex Sequencing, UMI-Based Methods)	Uses unique molecular identifiers (UMIs) to correct sequencing errors, improving accuracy	Focused on selected mutations	Ultra-high (10^−6^–10^−7^)	Detects ultra-low-frequency mutations, useful for MRD detection	High cost, requires advanced bioinformatics	Highly sensitive MRD monitoring, tracking clonal evolution, resistance detection

**Table 4:** Comparison of Illumina, Nanopore, and PacBio NGS Platforms for Leukemia Research.

Feature	Illumina	Oxford Nanopore	PacBio (SMRT Sequencing)
Sequencing Technology	Short-read sequencing (SBS—Sequencing by Synthesis)	Long-read sequencing using nanopores	Long-read sequencing using Single Molecule Real-Time (SMRT) technology
Read Length	Short reads (50–600 bp)	Ultra-long reads (up to >2 Mb)	Long reads (10–100 kb)
Accuracy	High (>99.9%)	Moderate (~90–98%)	High (~99.9%)
Error Profile	Low error rate, but struggles with structural variants and complex regions	Higher error rate, especially with homopolymers	Low error rate after consensus correction
Throughput	High (billions of reads per run)	Moderate (variable based on platform)	Moderate (lower than Illumina but higher than Nanopore)
Turnaround Time	Hours to days (depending on coverage)	Real-time sequencing (minutes to hours)	Hours to days
Cost per Base	Low ($)	Moderate ($$)	Higher than Illumina, but improving ($$$)
Best Applications in Leukemia	Targeted gene panels, whole-exome sequencing (WES), whole-genome sequencing (WGS), minimal residual disease (MRD) monitoring	Rapid real-time sequencing, structural variant detection, *BCR::ABL1* fusion identification	Structural variant detection, full-length transcript sequencing, clonal evolution studies
Strengths	High accuracy, cost-effective for large-scale sequencing, widely used in clinical applications	Real-time data output, ultra-long reads enable full-length fusion gene detection	High accuracy for long reads, superior for phasing and detecting epigenetic modifications
Limitations	Short reads may miss large structural variants, complex rearrangements	Higher error rate requires error correction, lower throughput than Illumina	Expensive, lower throughput than Illumina, requires high DNA quality

**Table 5:** Mutations and Variants Associated with ALL Identified by NGS.

Gene	Type of Mutation/Variant	Functional Impact	Clinical Significance
*ETV6::RUNX1*	Chromosomal translocation *t(12;21)*	Aberrant transcriptional regulation	Associated with a favorable prognosis, common in pediatric ALL
*BCR::ABL1*	Chromosomal translocation *t(9;22)* (Philadelphia chromosome)	Constitutive tyrosine kinase activation	Poor prognosis, treated with tyrosine kinase inhibitors (TKIs)
*CRLF2*	Overexpression due to translocations or mutations	Activates JAK-STAT signaling	Enriched in Ph-like ALL, associated with high risk
*IKZF1*	Deletions or point mutations	Loss of function in lymphoid differentiation	Poor prognosis, often co-occurs with Ph-like ALL
*PAX5*	Point mutations, deletions, translocations	Disrupts B-cell differentiation	Frequently mutated in B-ALL
*JAK1/JAK2*	Activating mutations	Constitutive cytokine signaling	Common in Ph-like ALL, potential target for JAK inhibitors
*FLT3*	Internal tandem duplication (ITD) or point mutations	Increased tyrosine kinase activity	Associated with high-risk ALL, possible target for FLT3 inhibitors
RAS Pathway (*NRAS*, *KRAS*, *PTPN11*)	Point mutations	Hyperactive RAS signaling	Contributes to chemoresistance and relapse risk
*NOTCH1*	Activating mutations	Dysregulation of T-cell development	Common in T-ALL, potential therapeutic target
*FBXW7*	Loss-of-function mutations	Increased NOTCH1 signaling	Associated with T-ALL, linked to resistance to therapy
*TP53*	Point mutations, deletions	Loss of tumor suppression	Poor prognosis, associated with therapy resistance
*CDKN2A/CDKN2B*	Deletions	Loss of cell cycle regulation	Common in both B-ALL and T-ALL, linked to poor prognosis
*EP300*, *CREBBP*	Loss-of-function mutations	Impaired histone acetylation and transcriptional regulation	Associated with resistance to corticosteroids and chemotherapy

**Table 6:** Mutations and Variants Associated with AML Identified by NGS.

Gene	Type of Mutation/Variant	Functional Impact	Clinical Significance
*FLT3*	Internal tandem duplication (ITD) or tyrosine kinase domain (TKD) mutations	Increased tyrosine kinase activity	Poor prognosis, associated with high relapse risk, targetable with FLT3 inhibitors (midostaurin, gilteritinib)
*NPM1*	Frameshift or insertion mutations	Aberrant cytoplasmic localization of nucleophosmin	Favorable prognosis in the absence of *FLT3-ITD*, common in younger AML patients
*IDH1/IDH2*	Point mutations	Alters DNA methylation and metabolism via 2-HG production	Targetable with IDH inhibitors (ivosidenib for *IDH1*, enasidenib for *IDH2*)
*DNMT3A*	Loss-of-function mutations	Aberrant DNA methylation and epigenetic dysregulation	Poor prognosis, associated with clonal hematopoiesis and therapy resistance
*TP53*	Point mutations, deletions	Loss of tumor suppression	Very poor prognosis, linked to therapy resistance and high relapse risk
*RUNX1*	Loss-of-function mutations	Disrupts hematopoietic differentiation	Associated with therapy-related AML and poor prognosis
*CEBPA*	Biallelic mutations	Impaired myeloid differentiation	Favorable prognosis if present in biallelic form
*KIT*	Activating mutations	Increased proliferation via RAS/MAPK signaling	Common in core-binding factor AML, worsens prognosis despite favorable karyotype
*TET2*	Loss-of-function mutations	Epigenetic dysregulation	Linked to clonal hematopoiesis, may contribute to resistance
*ASXL1*	Truncating mutations	Disrupts chromatin remodeling	Poor prognosis, frequently found in secondary AML
*WT1*	Frameshift or missense mutations	Defective tumor suppression	Associated with high relapse risk and poor prognosis
*RAD21*	Loss-of-function mutations	Impaired cohesin complex function	Contributes to genomic instability and therapy resistance
*SRSF2*	Splicing mutations	Abnormal RNA splicing	Common in secondary AML, often co-occurs with *RUNX1* and *ASXL1* mutations
*BCOR/BCORL1*	Loss-of-function mutations	Disrupts transcriptional repression	Associated with poor prognosis and resistance to therapy

**Table 7:** Mutations and Variants Associated with CLL Identified by NGS.

Gene	Type of Mutation/Variant	Functional Impact	Clinical Significance
*TP53*	Point mutations, deletions (*del(17p)*)	Loss of tumor suppression, genomic instability	Poor prognosis, resistance to chemoimmunotherapy, requires BTK or BCL2 inhibitors
*NOTCH1*	Frameshift or nonsense mutations	Constitutive activation of NOTCH signaling	Associated with Richter’s transformation, poor response to anti-CD20 therapy
*SF3B1*	Splicing mutations	Aberrant RNA splicing, altered gene expression	Linked to poor prognosis, resistance to fludarabine
*ATM*	Deletions (*del(11q)*) or point mutations	Impaired DNA repair	Associated with disease progression, sensitivity to PARP inhibitors
*BIRC3*	Loss-of-function mutations	Deregulated NF-κB signaling	Linked to resistance to chemoimmunotherapy, poor prognosis
*MYD88*	Activating mutations (L265P)	Hyperactive B-cell receptor (BCR) signaling	More common in atypical CLL, associated with better prognosis
*IGHV*	Hypermutation status (mutated or unmutated)	Affects BCR signaling dependency	Unmutated IGHV associated with more aggressive disease and poor response to chemotherapy
*XPO1*	Point mutations	Altered nuclear export of tumor suppressors	Associated with poor prognosis and therapy resistance
*EGR2*	Missense mutations	Impaired B-cell differentiation	Enriched in aggressive and relapsed CLL cases
*KRAS/NRAS*	Activating mutations	Increased RAS/MAPK signaling	Rare in CLL but linked to Richter’s transformation
*CARD11*	Gain-of-function mutations	Enhances BCR signaling	Associated with aggressive disease phenotypes
*FBXW7*	Loss-of-function mutations	Disrupts ubiquitin-mediated degradation of oncogenic proteins	Contributes to therapy resistance and disease progression
*DLEU2*	Deletions (part of *del(13q)*)	Loss of tumor suppressor miRNAs	Associated with favorable prognosis in isolated *del(13q)* cases

**Table 8:** Mutations and Variants Associated with CML Identified by NGS.

Gene/Fusion	Type of Mutation/Variant	Functional Impact	Clinical Significance
*BCR::ABL1* (Canonical)	Chromosomal translocation *t(9;22)* (Philadelphia chromosome)	Constitutive tyrosine kinase activation	Hallmark of CML, targetable with tyrosine kinase inhibitors (TKIs)
*BCR::ABL1* Kinase Domain Mutations	Point mutations (e.g., *T315I*, *E255K*, *Y253H*, *F317L*)	Resistance to TKIs	*T315I* mutation confers resistance to first- and second-generation TKIs, requiring ponatinib
Atypical *BCR::ABL1* Fusions	Alternative breakpoints in *BCR* or *ABL1* (e.g., *e19a2*, *e1a2*, *e6a2*)	Altered protein function and signaling activity	May affect TKI sensitivity and disease progression
Complex *BCR::ABL1* Fusion Variants	Involvement of third or additional partner genes (e.g., BCR::ABL1-ETV6, *BCR::ABL1-LAMP1*)	Additional oncogenic effects	May confer aggressive disease behavior or altered treatment responses
*ASXL1*	Loss-of-function mutations	Epigenetic dysregulation	Associated with disease progression and transformation to blast crisis
*RUNX1*	Point mutations, deletions	Impaired hematopoietic differentiation	Frequently mutated in blast phase CML, poor prognosis
*TET2*	Loss-of-function mutations	Alters DNA methylation and hematopoietic differentiation	May contribute to clonal evolution in advanced CML
*SETBP1*	Gain-of-function mutations	Enhances leukemic cell proliferation	Associated with progression to blast crisis and poor outcomes
*EZH2*	Loss-of-function mutations	Disrupts chromatin remodeling and gene silencing	Frequently mutated in advanced-phase CML, linked to aggressive disease
*IKZF1*	Deletions or loss-of-function mutations	Loss of lymphoid differentiation control	Predicts transformation to the lymphoid blast phase of CML
*NRAS/KRAS*	Activating mutations	Increases RAS/MAPK signaling, promoting leukemic cell survival, proliferation, and resistance to apoptosis.	Associated with TKI resistance and progression to blast crisis
*TP53*	Point mutations, deletions	Loss of tumor suppressor function	Poor prognosis, linked to disease progression and therapy resistance
*DNMT3A*	Loss-of-function mutations	Alters DNA methylation patterns	Implicated in clonal hematopoiesis and blast crisis transformation
*EVI1 (MECOM)*	Overexpression due to chromosomal rearrangements	Disrupts myeloid differentiation	Strongly associated with blast crisis and poor prognosis

**Table 9:** Molecular Goals of CML Treatment Based on qPCR.

Molecular Response (MR) *	*BCR::ABL1* Transcript Level (IS)	Clinical Significance
MR1	≤10%	Indicates early response; goal at 3 months to predict long-term outcomes
MR2	≤1%	Optimal response at 6 months; associated with improved survival
MR3 (Major Molecular Response, MMR)	≤0.1%	Standard treatment goal; associated with reduced risk of progression
MR4	≤0.01%	Indicates deep molecular remission (DMR); considered for treatment-free remission (TFR) evaluation
MR4.5	≤0.0032%	Deeper molecular response; increases the likelihood of successful TFR
MR5	≤0.001%	Undetectable disease by qPCR; associated with long-term remission and possible cure

*: The Molecular Response (MR) chart for CML was developed through consensus by international leukemia research groups, primarily led by the International CML Foundation (iCMLf), the European LeukemiaNet (ELN), and the National Comprehensive Cancer Network (NCCN). The standardization of molecular response levels was established using the International Scale (IS) for *BCR::ABL1* quantification, which was introduced in 2006 by the International Randomized Study of Interferon vs. STI571 (IRIS) trial in collaboration with the World Health Organization (WHO). The ELN guidelines first formalized these molecular milestones, defining MR3 (MMR), MR4, and MR4.5 as key targets for treatment success and eligibility for treatment-free remission (TFR). The National Comprehensive Cancer Network (NCCN) and WHO have since integrated these MR levels into their global CML management recommendations. The MR5 category, while widely recognized, is less frequently used in clinical practice due to limitations in standard qPCR sensitivity.

## Data Availability

No new data were created.
